# Novel easily available purine-based AIEgens with colour tunability and applications in lipid droplet imaging†
†Electronic supplementary information (ESI) available. CCDC 1855630–1855632 and 1855634. For ESI and crystallographic data in CIF or other electronic format see DOI: 10.1039/c8sc03369b


**DOI:** 10.1039/c8sc03369b

**Published:** 2018-09-19

**Authors:** Lei Shi, Kun Li, Ling-Ling Li, Shan-Yong Chen, Meng-Yang Li, Qian Zhou, Nan Wang, Xiao-Qi Yu

**Affiliations:** a Key Laboratory of Green Chemistry and Technology , Ministry of Education , College of Chemistry , Sichuan University , Chengdu , China 610064 . Email: kli@scu.edu.cn ; Email: xqyu@scu.edu.cn

## Abstract

Recently, tetraphenylethene, triphenylamine and other man-made core AIE luminescent materials (AIEgens) have attracted significant scientific interest. However, the design and synthesis of natural product based, facile and color tunable AIEgens remains challenging. Herein, a novel series of AIEgens based on purine-core molecular rotors is reported, which can be facilely synthesized and shows color tunable emission. Moreover, these purine-based AIEgens exhibit lipid droplet specific properties in live cellular imaging with low background, high selectivity and excellent biocompatibility.

## Abstract 2

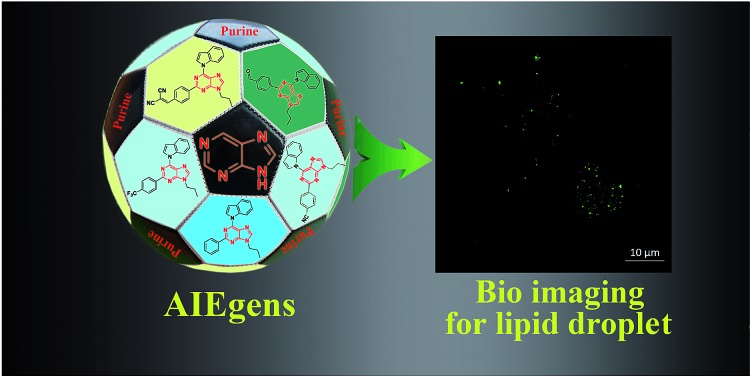
A new series of purine-based derivatives with easy availability and colour tunability were synthesized and could be used for lipid droplet imaging.

## Introduction

Since the concept of aggregation-induced emission (AIE) was established in 2001,^1^ fluorophores with AIE characteristics (AIEgens) have experienced rapid expansion in the diversity of molecules,^2^ mechanistic understanding^3^ and applications.^4^ Nowadays, the design and synthesis of AIEgens with bright and tunable emission have attracted significant scientific interest.^5^ Among various AIEgens, the restriction of intramolecular motion (RIM) as a more general mechanism has been widely used in design strategies.^6^ Compared with traditional organic fluorophores, AIEgens based on the RIM mechanism exhibit many advantages such as high brightness, excellent photostability in the aggregated state and low fluorescence in the dissolved state.^7^ On the other hand, in cellular imaging, there are many fluorescent dyes designed to accumulate in specific organelles. It is worth noting that, as for traditional dyes, accumulation could cause a concentration quenching effect which leads to fluorescence quenching.^8^ However, as for AIEgens, the aggregation process could cause bright fluorescence and colour tunability, which is highly desirable for imaging of subcellular organelles such as mitochondria,^9^ lysosomes,^10^ cell membranes^11^ and lipid droplets.^12^

Lipid droplets (LDs) are mainly found in adipocytes, the adrenal cortex and hepatocytes, which mainly contain three categories of neutral lipids such as glycerol and cholesterol esters.^12^ In the past few years, LDs have been regarded as a form of neutral lipid storage in inert reservoirs. However, recent studies show that LDs are thought to be dynamic organelles, and are related to lipid storage and metabolism, signal transduction and apoptosis.^13^ LDs are closely related to many metabolic processes and diseases, such as membrane lipid flow,^14^ viral infection,^15^ inflammation,^16^ cancer^17^ and obesity.^18^ Therefore, the localization and analysis of LDs are of great importance for biomedical research and clinical diagnosis.

**Fig. 1:**
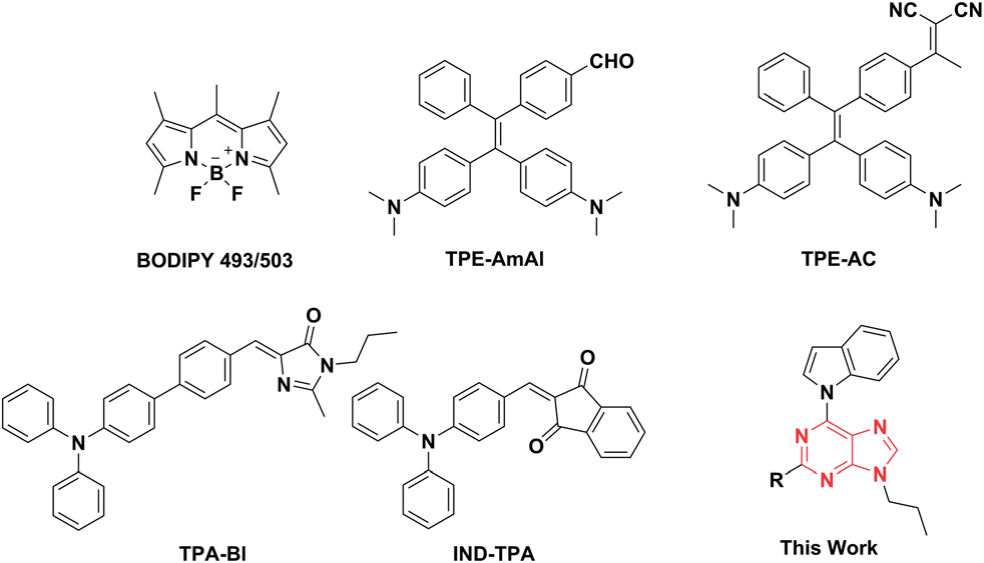
Chemical structures of lipid droplet imaging probes.

Although many fluorescent probes for LD localization have been previously reported, most of them have exhibited many drawbacks, such as strong background fluorescence, small Stokes shifts^19^ and ACQ (aggregation-caused quenching) effects, which will result in incomplete and inaccurate biological imaging. Several LD-specific AIE bioprobes have been designed and synthesized to solve these problems. Indeed, LD-specific AIE bioprobes, such as **TPE-AmAl**, **TPE-AC**, **TPA-Bl**, and **IND-TPA** (Fig. 1), show better performances in brightness, specificity, and photostability than BODIPY 493/503 in living cellular imaging. Recently, the Tang group and the Klymchenko group reported several AIEgens used for LD imaging which emit in the red region and even the NIR region with high brightness.12d–f However, these AIEgens are merely based on tetraphenylethene (TPE) and triphenylamine (TPA), which belong to a group of manually designed and synthesized molecules. Development of new core structures to expand the varieties of AIEgens, especially with prominent biocompatibility and high brightness, is therefore necessary for further applications in LD targeted cell imaging. When preparing this manuscript, we found that the Tang group had recently explored natural isoquinoline alkaloids with superior AIEgen properties.^20^ Herein, we innovatively adopted purine as the core of our AIEgens and synthesized a series of new compounds with different electron donor/acceptor abilities, expecting them to have good AIE performances and cell-targeting imaging abilities.

Purine, a component of DNA and RNA when combined with specific pyrimidine bases, is a vital chemical in natural organisms. In recent years, purine chemistry has developed rapidly due to the biological importance of purine and the research of pharmaceutical chemists in the fight against tumours and in antiviral drugs.^21^ Besides, due to its large π-conjugated plane, purine is an excellent core structure for fluorescent probes. To the best of our knowledge, purine has never been used to construct fluorescent probes. Therefore, we decided to adopt purine as the core structure to build a series of AIE fluorophores with a tunable emission colour which could be used for LD targeted cell imaging. For the building of tunable emission fluorophores, it is better to increase the molecular conjugation length or choose a decent combination of an electron donor (D) and an electron acceptor (A). To avoid ACQ effects and simplify the difficulty of the synthesis, a D–π–A structure is an idea model.^22^ Thus, we chose cheap and easily available indole to be the electron donor and changed the electron acceptor to regulate the emission. Meanwhile, we introduced an *n*-propyl group to the fluorophore to improve its fat solubility.

## Results and discussion

**Fig. 2:**
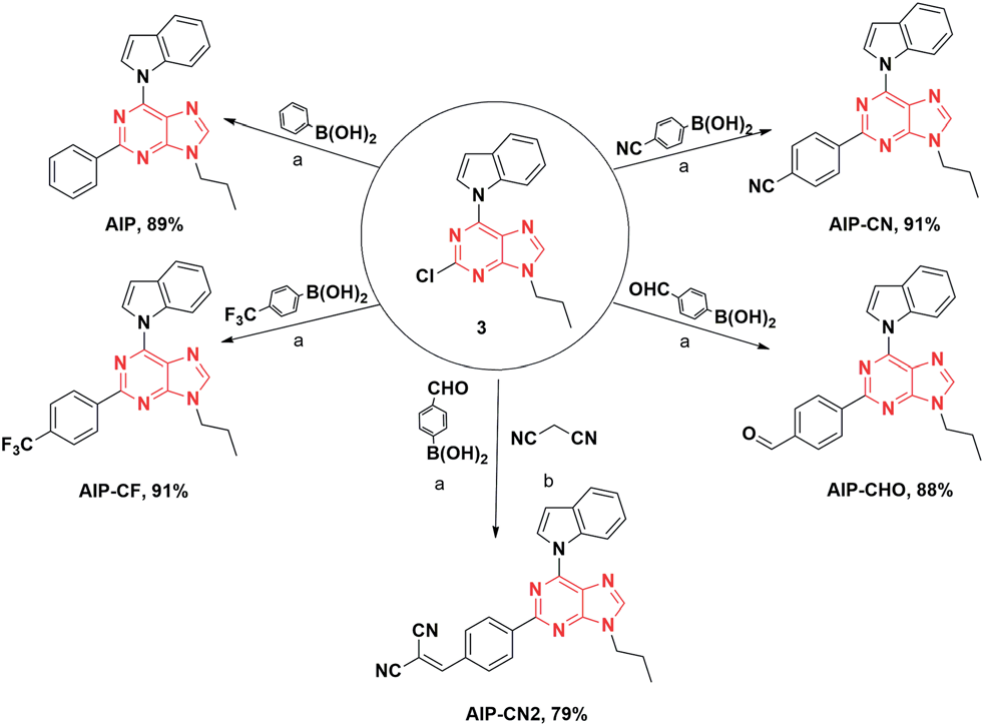
Synthetic routes to **AIP**, **AIP-CF**, **AIP-CN**, **AIP-CHO** and **AIP-CN2**. Reagents and conditions: (a) Pd(PPh_3_)_4_, K_2_CO_3_, 2,6-dioxane, water, 80 °C, 8 h; (b) malononitrile, DMF, piperidine, room temperature, 2 h.

The initial step of our research was to merge the components of the acceptor to form the desired compounds (Fig. 2). After combining with indole and propyl, all purine derivatives, including **AIP**, **AIP-CF**, **AIP-CN**, and **AIP-CHO**, were synthesized through Suzuki coupling reactions. Additionally, **AIP-CN2** was prepared from the starting materials for **AIP-CHO** with the addition of malononitrile. All the compounds were easily purified *via* column chromatography with reasonable yields. All of the target products were characterized by ^1^H NMR, ^13^C NMR, and high resolution mass spectrometry, which confirmed their correct structures.

The absorption and photoluminescence properties of all compounds in solution, aggregation and the solid state are shown in Table 1, as well as Tables S1 and S2.† Each compound has a high absorption peak that appears around *λ* = 330 nm which is attributed to a π–π* transition. When the substituent group at the 2-position was altered from phenyl to *o*-(2-methylenemalononitrile)-phenyl, the corresponding compounds exhibited the longest wavelength emission peak ranging from 395–533 nm, which was assigned to the ICT transition from the electron-donating indole group to the different types of electron acceptor groups.^23^ Additionally, a similar red shifted emission trend for the solid state was observed (from 441 to 522 nm).

**Table 1:** Optical properties of all compounds

AIEgens	*λ*_abs_ ^*a*^ (nm)	*ε* ^*a*^ (M^–1^ cm^–1^)	*λ*_em_ (nm)			*α*_AIE_ (*I*_aggr,max_/*I*_soln_)	*τ*_avg_ ^*e*^ (s)
			Soln (*Φ*_F_)^*b*^	Aggr (*Φ*_F_)^*c*^	Solid (*Φ*_F_)^*d*^		
**AIP**	330	5.70 × 10^5^	424 (87.6)	404 (23.6)	441 (33)	0.7	5.44 × 10^–10^
**AIP-CF**	328	4.02 × 10^5^	426 (10.9)	424 (26.4)	450 (93.5)	6.8	4.11 × 10^–9^
**AIP-CN**	316	5.88 × 10^5^	450 (2.2)	434 (37.9)	473 (80.2)	36.8	8.33 × 10^–9^
**AIP-CHO**	316	7.80 × 10^5^	410 (1.3)	462 (5.1)	491 (7.3)	80.1	1.53 × 10^–2^
**AIP-CN2**	328	9.33 × 10^5^	n.d. (0.7)	548 (5)	522 (10.2)	39.2	5.53 × 10^–3^

^*a*^Absorption maximum in DMSO.

^*b*^Emission maximum of the solution state in DMSO.

^*c*^Emission maximum of the aggregation state in PBS.

^*d*^Emission maximum in the solid state.

^*e*^Fluorescence lifetime, measured under ambient conditions. Fluorescence quantum yield determined by a calibrated integrating sphere.

**Fig. 3:**
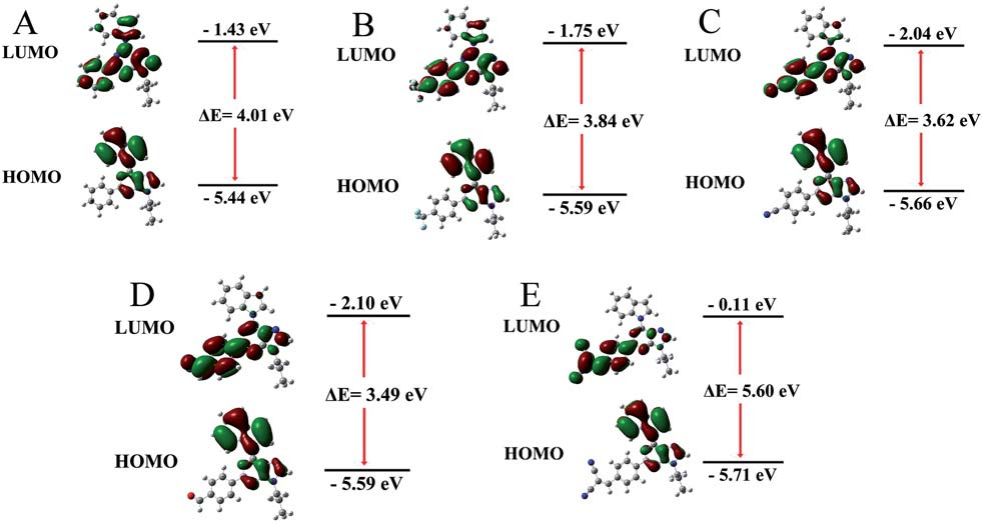
Molecular orbital amplitude plots of HOMO and LUMO energy levels of (A) **AIP**, (B) **AIP-CF**. (C) **AIP-CN**, (D) **AIP-CHO** and (E) **AIP-CN2** calculated by using the B3LYP/6-31G (d,p) basis set.

**Fig. 4:**
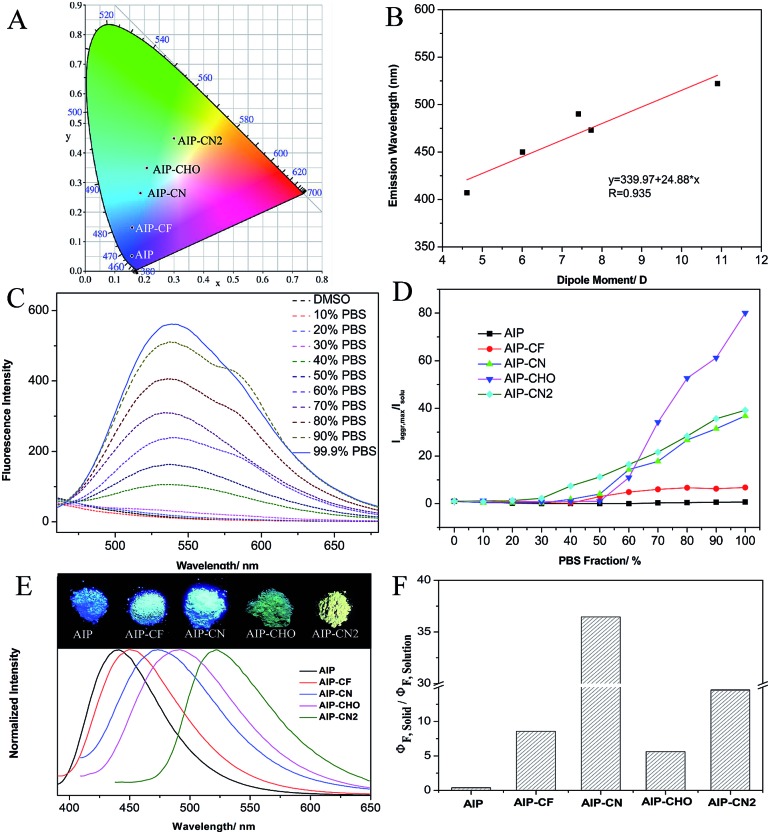
(A) Solid fluorescence spectra of all compounds plotted on a CIE 1931 chromaticity diagram; (B) the linear correlation of the maximum emission fluorescence wavelength and the dipole moment; (C) fluorescence spectra of **AIP-CN2** in DMSO/PBS with different PBS fractions; (D) variation in relative fluorescence intensity (*I*/*I*_0_) of all compounds in DMSO/PBS with different PBS fractions; (E) normalized fluorescence spectra of all compounds as solids, and photographs of the fluorescence of all compounds taken under illumination with UV light (*λ* = 365 nm); (F) the ratio of the quantum yields for the solid and solution states of all compounds.

To better understand the optical behaviors of these purine-based AIEgens, density functional theory (DFT) calculations were performed. The ground state geometries of all compounds were optimized using DFT with B3LYP hybrid functions at the basis-set level of 6-31G*. As shown in Fig. 3, the calculation results showed that the majority of the electron distribution of the highest occupied molecular orbital (HOMO) was very similar for each compound and was located on the indole moiety which belongs to the electron donor group. The lowest unoccupied molecular orbital (LUMO) is more delocalized over the electron acceptor group with increasing electrical attraction. These results show the D–A structural features of **AIP**, **AIP-CF**, **AIP-CN**, **AIP-CHO** and **AIP-CN2**. The CIE chromaticity diagrams (Fig. 4A and S6†) demonstrated the same tunable emission from CIE blue (0.1574, 0.06) to CIE green (0.3004, 0.4490).

To evaluate the effect of the electrical attraction of different electron acceptor groups on the fluorescence behaviour of these purine-based derivatives, the change in the maximum fluorescence wavelength and the dipole moment was plotted in Fig. 4B. The linear correlation with the correlation coefficient *R* = 0.935 and the slope of 24.88 show that the different electron acceptor groups have significant influences on the purine derivatives, which can guide us to predict the emission wavelengths of purine-based derivatives by calculation.

Additionally, these five compounds exhibit remarkable AIE properties (Fig. 4C). We measured the AIE properties using DMSO and phosphate buffer saline (PBS), which served as a good and poor solvent, respectively. For example, while **AIP-CN2** is almost non-emissive in DMSO, the aggregations of **AIP-CN2**in the DMSO/PBS (>60% phosphate buffer saline) are highly emissive. The observed phenomenon could be attributed to aggregation of the molecules in the mixed solution with a high *f*_P_, and thus activation of the RIM process. The compounds **AIP-CHO**, **AIP-CN**, and **AIP-CF** exhibited similar phenomena to **AIP-CN2**. Notably, the phenomenon that the fluorescence intensity of **AIP** decreases first and is then enhanced with the increase of PBS was caused by TICT and AIE properties. Incidentally, when the PBS fraction is increased to 99.9%, the fluorescence intensities of **AIP**, **AIP-CF**, **AIP-CN**, **AIP-CHO**, and **AIP-CN2** are about 0.7, 6.8, 36.8, 80.1, and 39.2 times greater than those in pure DMSO, respectively (Fig. 4D). The emission behaviours of all of the compounds in the solid state were also studied. The photographs of the solids under irradiation at 365 nm and the fluorescence spectra of all compounds are given in Fig. 4E. The emission intensities are much stronger than those in the PBS mixtures apart from that of **AIP**.

The fluorescence quantum yields (*Φ*_F_) of all compounds in solutions, in aggregations, and in the solid state are summarized in Table 1. The quantum yields of **AIP-CF**, **AIP-CN**, **AIP-CHO** and **AIP-CN2** in the solid state (93.5%, 80.2% 7.3% and 10.2%, respectively) were relatively higher compared with those when dissolved in DMSO (10.9%, 2.2% 1.3% and 0.7%, respectively). In contrast, a poor AIE phenomenon for **AIP** was observed in the solid state (33.0%) compared with that in solution (87.6%). We also calculated the ratio between the solid and solution state quantum yields to verify the influence of the substituent group on the emission behaviours of the compounds (Fig. 4F).

The fluorescence lifetime (*τ*) values of all the fluorophores are listed in Tables 1 and S4†. Interestingly, we found that the fluorescence lifetimes of **AIP-CHO** and **AIP-CN2** in the solid state reached 15.3 and 5.5 ms, respectively, which means that these two compounds had delayed fluorescence properties for the long fluorescence lifetime.^24^ To verify the photoemission dynamics, the radiative transition rate constant (*k*_r_) as well as the non-radiative transition rate constant (k_nr_) were calculated using the fluorescence lifetimes and the quantum yields (see Table S6†).

**Fig. 5:**
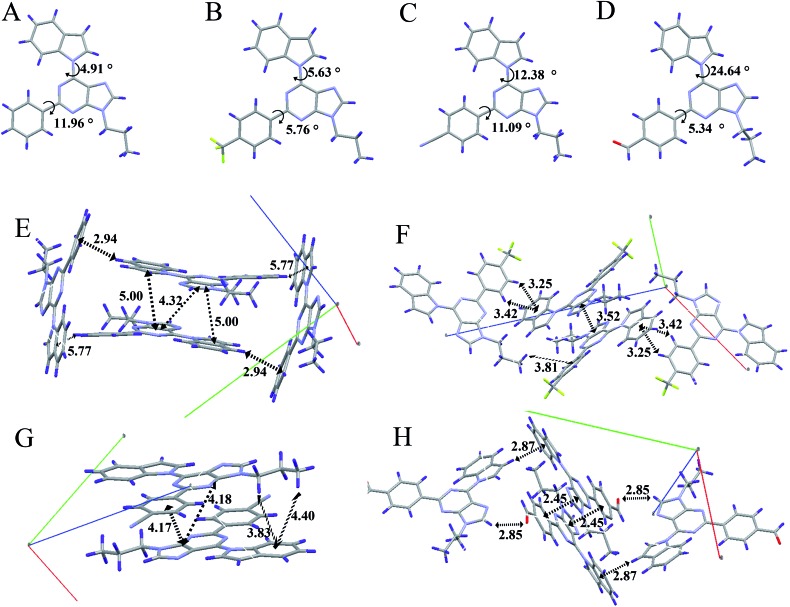
Single-crystal structures of (A) **AIP**, (B) **AIP-CF**, (C) **AIP-CN** and (D) **AIP-CHO**; and the molecular packing of (E) **AIP**, (F) **AIP-CF**, (G) **AIP-CN** and (H) **AIP-CHO**. Carbon, hydrogen, nitrogen and oxygen atoms are shown in grey, blue, mauve, and red, respectively.

In order to understand the AIE properties of the purine-based derivatives and the changes in their emission colours caused by different electron acceptor groups, we have studied single crystal structures to reveal their molecular conformations and crystal packing patterns. The single crystal structures of **AIP**, **AIP-CF**, **AIP-CN**, and **AIP-CHO** are shown in Fig. 5 and S10.† In general, while the twist angles between the purine core and the electron acceptor groups show minor changes ranging from 5.34°to 11.96°, the different electron acceptor groups caused significant transformations to the twist angles between the purine core and the electron donor group. With increasing electronic affinity of the acceptor, the twist angles between the purine core and the donor group gradually increased, ranging from 4.91° to 24.64°. The twist angles and the changes of dipole moment may influence the maximum fluorescence wavelength of these purine-based derivative. Besides, the packing modes show that **AIP**, **AIP-CF**, and **AIP-CN** stack in the form of head-to-tail and no strong intermolecular interactions are observed except for some weak interactions such as C–H···π and C–H···N. Moreover, with an increase of a molecule’s dipole interaction, the distance between the adjacent molecules significantly reduces as shown in Table S7.† Among these four compounds, **AIP-CHO** due to its strong C–H···O interaction shows a shorter distance than **AIP-CN**. According to previous research,^25^ there is no π–π stacking when the distance is larger than 3.7 Å. Therefore, the low fluorescence quantum yields of **AIP-CHO** may be attributed to its short distance between two molecules of 2.45 Å which is caused by the strong dipole interaction and the C–H···O interaction. Unfortunately we have not solved the crystal structure of **AIP-CN2**, but we guess its low quantum yield may be caused by the same π–π interactions due to the short distance between two molecules.

To explore the application of all compounds in LD imaging in live cells, the cytotoxicity of all compounds was firstly evaluated using a 3-(4,5-dimethylthiazol-2-yl)-5-(3-carboxymethoxyphenyl)-2-(4-sulfophenyl)-2*H*-tetrazolium (MTS) assay. As shown in Fig. S11,† there was no significant change in the cell viability when the cell were cultured with different concentrations of the probes for 24 h. This reveals that the purine-based probes show no cytotoxicity and have good biocompatibility with live cells.

Then, we investigated their capability to stain LDs in live cells by confocal lasing scanning microscopy (CLSM). In order to evaluate the specificity of these purine-based probes towards LDs in live HeLa cells, a commercially available LD-imaging agent BODIPY 493/503 was used to co-stain the HeLa cells. To avoid optical crosstalk, we excited the longest emission-wavelength probe **AIP-CN2** and BODIPY 493/503 at 405 and 488 nm, respectively. We measured the emission of **AIP-CN2** from 420 to 480 nm, and BODIPY 493/503 from 530 to 560 nm, respectively. As shown in Fig. S12,† no optical crosstalk was observed.

**Fig. 6:**
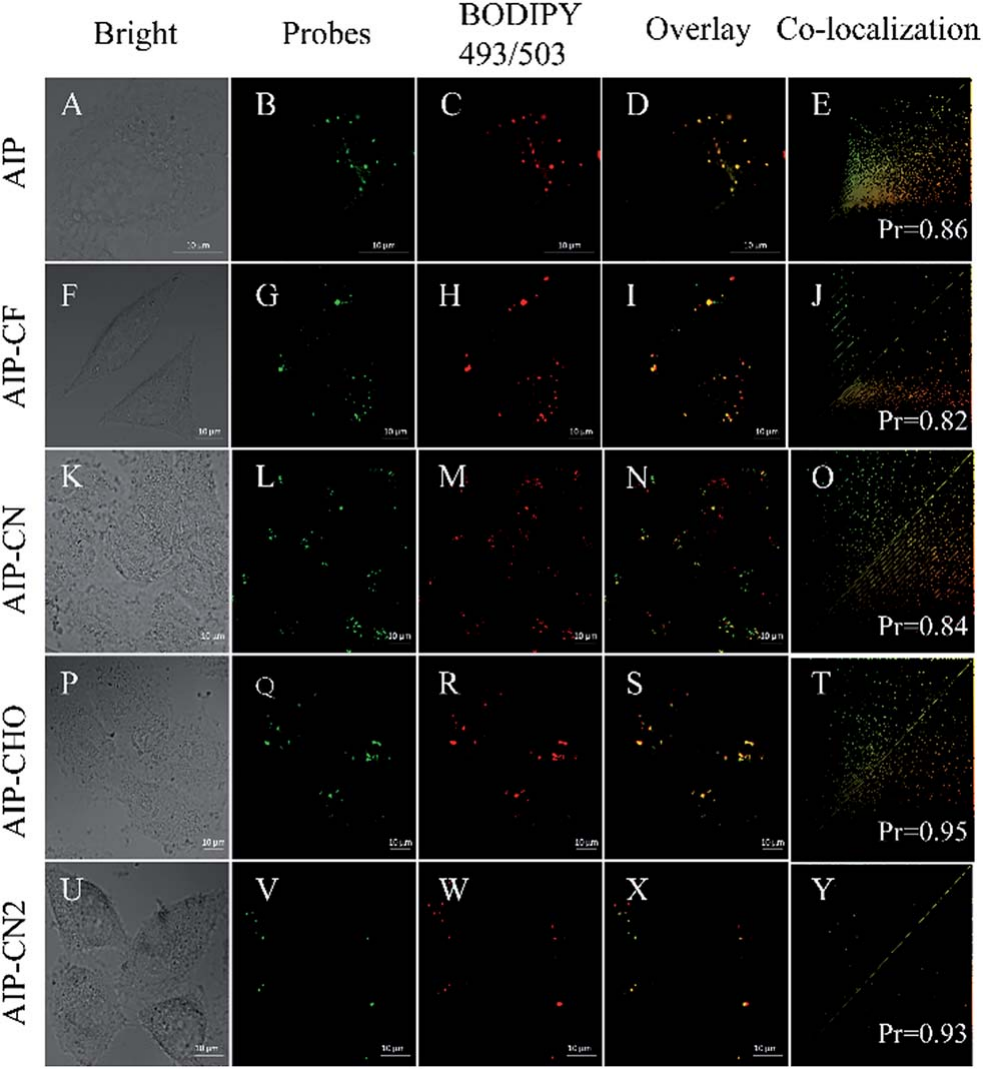
Live HeLa cells incubated with BODIPY 493/503 and (A–E) **AIP**, (F–J) **AIP-CF**, (K–O) **AIP-CN**, (P–T) **AIP-CHO**, and (U–Y) **AIP-CN2**. Probe Channel, *λ*_ex_ = 420 nm, and *λ*_em_ = 420–480 nm. BODIPY 493/503 channel, *λ*_ex_ = 488 nm and *λ*_em_ = 530–560 nm.

Subsequently, cell imaging experiments were carried out by incubating HeLa cells with probes (*λ*_ex_ = 405 nm) and BODIPY 493/503 (*λ*_ex_ = 488 nm) successively. As shown in Fig. 6, the fluorescence of **AIP**, **AIP-CF**, **AIP-CN**, **AIP-CHO** and **AIP-CN2** channel together with the BODIPY 493/503 channel are pretty well co-related. Pearson correlation coefficients of the purine-based AIEgens and BODIPY 493/503 were calculated in the range of 0.82 to 0.95, indicating high selectivity for LDs. Meanwhile, the photostability experiments indicated that the photostabilities of these probes were similar to those of commercial dyes (Fig. S13†). These results suggested that the present purine-based AIEgens could serve as novel AIE dyes for lipid droplets in cellular imaging.

## Conclusions

In summary, we successfully designed and synthesized a series of novel purine-based AIEgens with LD-specific properties. Their optical properties and theoretical calculations as well as single crystal packing structures were systematically investigated. The results showed that purine is an ideal core structure for building easily available AIEgens with color tunability. Additionally, these probes displayed good cell biocompatibility, high brightness, low background, high selectivity and a wide luminous range. These combined characteristics of the purine-based derivatives demonstrated the potential of the novel AIE fluorophores for biological applications.

## Conflicts of interest

There are no conflicts to declare.

## Supplementary Material

Supplementary information

Crystal structure data

## References

[cit1] Luo J., Xie Z., Lam J., Cheng L., Chen H., Qiu C., Kwok H. S., Zhan X., Liu Y., Zhu D., Tang B. Z. (2001). Chem. Commun..

[cit2] Shi L., Li K., Cui P. C., Li L. L., Pan S. L., Li M. Y., Yu X. Q. (2018). J. Mater. Chem. B.

[cit3] Qian H., Cousins M. E., Horak E. H., Wakefield A., Liptak M. D., Aprahamian I. (2017). Nat. Chem..

[cit4] Xu S., Liu T., Mu Y., Wang Y. F., Chi Z., Lo C. C., Liu S., Zhang Y., Lien A., Xu J. (2015). Angew. Chem., Int. Ed..

[cit5] Peng Q., Obolda A., Zhang M., Li F. (2015). Angew. Chem., Int. Ed..

[cit6] Mei J., Hong Y., Lam J. W. Y., Qin A., Tang Y., Tang B. Z. (2014). Adv. Mater..

[cit7] Ding D., Li K., Liu B., Tang B. Z. (2013). Acc. Chem. Res..

[cit8] Tansi F. L., Rüger R., Rabenhold M., Steiniger F., Fahr A., Kaiser W. a., Hilger I. (2013). Small.

[cit9] Zhao N., Chen S., Hong Y., Tang B. Z. (2015). Chem. Commun..

[cit10] Hu F., Cai X., Manghnani P. N., Kenry, Wu W., Liu B. (2018). Chem. Sci..

[cit11] Wang D., Su H., Kwok R. T. K., Hu X., Zou H., Luo Q., Lee M. M. S., Xu W., Lam J. W. Y., Tang B. Z. (2018). Chem. Sci..

[cit12] Kang M., Gu X., Kwok R. T. K., Leung C. W. T., Lam J. W. Y., Li F., Tang B. Z. (2016). Chem. Commun..

[cit13] Fujimoto T., Ohsaki Y., Cheng J., Suzuki M., Shinohara Y. (2008). Histochem. Cell Biol..

[cit14] Zehmer J. K., Huang Y., Peng G., Pu J., Anderson R. G. W., Liu P. (2009). Proteomics.

[cit15] Lyn R. K., Kennedy D. C., Stolow A., Ridsdale A., Pezacki J. P. (2010). Biochem. Biophys. Res. Commun..

[cit16] Bozza P. T., Viola J. P. (2010). Prostaglandins, Leukotrienes Essent. Fatty Acids.

[cit17] Tirinato L., Liberale C., Di Franco S., Candeloro P., Benfante A., La Rocca R., Potze L., Marotta R., Ruffilli R., Rajamanickam V. P., Malerba M., De Angelis F., Falqui A., Carbone E., Todaro M., Medema J. P., Stassi G., Di Fabrizio E. (2015). Stem Cells.

[cit18] Nagayama M., Uchida T., Gohara K. (2007). J. Lipid Res..

[cit19] Greenspan P., Mayer E. P., Fowler S. D. (1985). J. Cell Biol..

[cit20] Gu Y., Zhao Z., Su H., Zhang P., Liu J., Niu G., Li S., Wang Z., Kwok R. T. K., Ni X. L., Sun J., Qin A., Lam J. W. Y., Tang B. Z. (2018). Chem. Sci..

[cit21] Demange L., Abdellah F. N., Lozach O., Ferandin Y., Gresh N., Meijer L., Galons H. (2013). Bioorg. Med. Chem. Lett..

[cit22] Nan Zhang J., Kang H., Li N., Zhou S. M., Sun H. Mi., Yin S. W., Zhao N., Tang B. Z. (2017). Chem. Sci..

[cit23] Grabowski Z. R., Rotkiewicz K., Rettig W. (2003). Chem. Rev..

[cit24] Xie Z., Chen C., Xu S., Li J., Zhang Y., Liu S., Xu J., Chi Z. (2015). Angew. Chem., Int. Ed..

[cit25] Xu S., Liu T., Mu Y., Wang Z., Chi Z., Lo C., Liu S., Zhang Y., Alan L., Xu J. (2015). Angew. Chem., Int. Ed..

