# Supplementary material for: Novel easily available purine-based AIEgens with colour tunability and applications in lipid droplet imaging
Source: Chem Sci. 2018 Sep 19;9(48):8969–74. doi: 10.1039/c8sc03369b (PMC6301202; doi:10.1039/c8sc03369b)
Supplement: Supplementary file 1 [file SC-009-C8SC03369B-s001.pdf]

Electronic Supplementary Material (ESI) for Chemical Science.
This journal is © The Royal Society of Chemistry 2018

Supporting Information for

Novel Easily Available Purine-Based AIEgens with Color Tunability and Application of Lipid Droplets Imaging

Lei Shi, Kun Li,* Ling-Ling Li, Shan-Yong Chen, Meng-Yang Li, Qian Zhou, Nan Wang, and Xiao-Qi Yu*

Key Laboratory of Green Chemistry and Technology, Ministry of Education, College of Chemistry, Sichuan University, Chengdu, China 610064

E-mail: kli@scu.edu.cn; xqyu@scu.edu.cn

Received Date (will be automatically inserted after manuscript is accepted)

Contents

1. Experimental section S2
2. Molar Extinction Coefficient of all compounds……...S6
3. Solvent effect of all compounds..S7
4. Fluorescent spectra of all compounds in DMSO/PBS mixtures S9
5. Particle size of all compounds in aggregation state..S11
6. Solid fluorescence spectra of all compounds and their CIE diagrams……………S12
7. Fluorescent lifetime and quantum yield of all compounds in solution, in aggregation and in the solid state...S15
8. Theoretical Calculation of all compounds..S16
9. Views of the molecular stacking structures in single crystals of AIP, AIP-CF, AIP-CN and AIP-CHO..S18
10. Crystallographic data of AIP, AIP-CF, AIP-CN and AIP-CHO……..………S21
11. Cytotoxicity of all compounds on Hela cells evaluated by MTS assay S22
12. Single-photo CLSM images of Hela cells incubated with all compounds and BODIPY 493/503..S22
13. Photostability of all compounds in CLSM imaging……………………………S22
14. NMR Data...S23
15. ESI-HRMS Data S29

1. Experimental Section

1.1 General information

All chemicals and solvents were commercially available and were used without further purification. 2,6-dichloropurine, 1-bromopropane, indole, 4-formylphenylboronic acid, 4-cyanophenylboronicacid and (4-(Trifluoromethyl)phenyl)boronic acid were purchased from Innochem. ^{1} H NMR, ^{13}C NMR spectra were measured on a Bruker AM400 NMR spectrometer. Proton Chemical shifts of NMR spectra were given in ppm relative to internals reference TMS (1H, 0.00 ppm). ESI-HRMS spectral data were recorded on a Finnigan LCQDECA mass spectrometer. Fluorescence emission spectra were obtained using Hitachi F-7000 spectrometer at 298 K. Absorption spectra were recorded on a Hitachi PharmaSpec UV-1900 UV-Visible Spectrophotometer. The absolute fluorescence quantum yield was measured using a Hamamatsu quantum yield spectrometer C11347 Quantaurus_QY. The fluorescence lifetime was measured using a Hamamatsu Compact Fluorescence Lifetime Spectrometer C11367. Single crystal were grown from petroleum ether/dichloroform via solute solution diffusion method. Single crystal X-ray diffraction intensity data were collected on Agilent Technologies (Gemini). The ground-state geometries were optimized using the density function theory (DFT) method with B3LYP hybrid functional at the basis set level of 6-31G (d, p). All the calculations were performed using Gaussian 09 package. MTS method was used for testing the cell viability and described in the experimental section. HeLa cells were obtained from Shanghai Institute of Biochemistry and Cell Biochemistry and Cell Biology, Chinese Academy of Science. Confocal lasing scanning microscopic (CLSM) images of single-photo were obtained using LSM 780 (Zeiss). Unless otherwise noted, materials were obtained from commercial suppliers and were used without further purification. All the solvents were dried according to the standard methods prior to use. All of the solvents were either HPLC or spectroscopic grade in the optical spectroscopic studies.

1.2 Reaction procedures

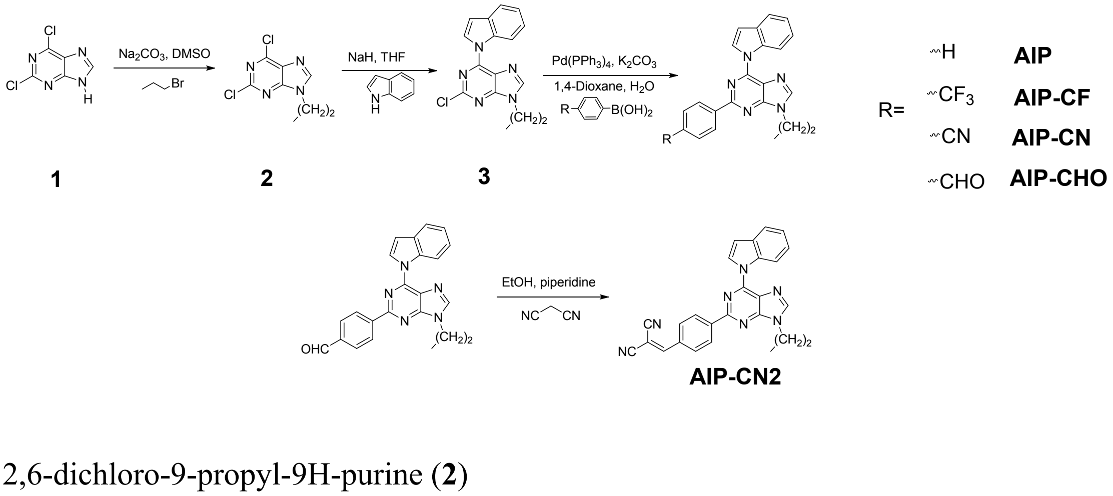

2,6-dichloro-9-propyl-9H-purine (**2**)

A mixture of 2,6-dichloropurine **1** (1.0 mmol), 1-bromopropane (1.5 mmol), and potassium carbonate (3.0 mmol) in DMSO (5 mL) was stirred for 6 h, then the mixture was filtered and evaporated under vacuum. The products were separated by flash chromatography on silica gel eluting with EtOAc/DCM (2:3) as white solid in 61% yield. ^{1}H NMR (400 MHz, $CDCl_3$): δ (TMS, ppm) 8.10 (s, 1H), 4.19 (t, J = 7.2 Hz, 2H), 1.90 (q, J = 7.3 Hz, 2H), 0.91 (t, J = 7.4 Hz, 3H).

2-chloro-6-(1H-indol-1-yl)-9-propyl-9H-purine (**3**)
Under nitrogen, indole (1g, 14 mmol) was added to a suspension of NaH (2 g, 21 mmol, 60% dispersion in mineral oil) in dry THF (500 mL) at 0 °C with stirring. The resulting solution was stirred at 0 °C for 1 h, and then compound **2** (3.2 mL, 14 mmol, resolved in 50 mL dry THF) was slowly added. The mixture was allowed to warm to room temperature and stirred overnight. Water was added to quench the reaction. The organic layer was separated, and the aqueous layer was extracted with ethyl acetate (30 mL × 3). The combined organic extracts were washed with brine, and dried over Na_2SO_4. After removal of the solvent, the residue was purified by column chromatography on silica gel. Elution with hexane/ethyl acetate (3:1) gave compound **3** as a white solid in 57% yield. ^{1}H NMR (400 MHz, $CDCl_3$): δ (TMS, ppm) 9.12 (s, 1H), 8.95 (d, J = 8.4 Hz, 1H), 7.95 (s, 1H), 7.61 (d, J = 7.7 Hz, 1H), 7.39 (t, J = 7.3 Hz, 1H), 7.29 (t, J = 7.1 Hz, 1H), 6.79 (d, J = 3.7 Hz, 1H), 4.20 (t, J = 7.3 Hz, 2H), 1.95 (q, J = 7.3 Hz, 2H), 0.99 (d, J = 14.8 Hz, 3H).

6-(1H-indol-1-yl)-2-phenyl-9-propyl-9H-purine (**AIP**)
Compound **3** (342 mg, 1.1 mmol), phenylboronic acid (1.5 eq), $Pd(PPh_3)_4$ (0.05eq) and 2.0 mL Na_2CO_3 solution (2M) in 10.0 mL 2,6-dioxane was refluxed for 8 hrs under N_2, After the reaction was completed based on the TLC, poured the reaction mixture into water and extracted with DCM. The organic layer was washed with brine, water and dried over anhydrous Na_2SO_4. The crude product was purified by column chromatography on silica gel. Elution with DCM gave **AIP** as a white solid in 89% yield. ^{1}H NMR (400 MHz, d^{6}-DMSO): δ (TMS, ppm) 9.20 (d, J = 3.7 Hz, 1H), 9.04 (d, J = 8.4 Hz, 1H), 8.63 (s, 1H), 8.52 – 8.46 (m, 2H), 7.69 (d, J = 7.6 Hz, 1H), 7.59 (t, J = 7.2 Hz, 2H), 7.53 (t, J = 7.2 Hz, 1H), 7.42 (t, J = 7.2 Hz, 1H), 7.28 (t, J = 7.0 Hz, 1H), 6.91 (d, J = 3.7 Hz, 1H), 4.30 (t, J = 7.1 Hz, 2H), 1.94 (q, J = 7.2 Hz, 2H), 0.90 (t, J = 7.4 Hz, 3H). ^{13}C NMR (100 MHz, d^{6}-DMSO): δ (TMS, ppm) 157.85, 154.06, 149.01, 145.89, 138.09, 135.65, 130.86, 130.60, 129.27, 128.95, 128.26, 124.33, 123.17, 121.46, 121.00, 116.73, 108.45, 45.42, 23.03, 11.50. HRMS (ESI): *m/z*: Calcd for $C_{22}H_{20}N_5^+$: 354.1718 $[M+H]^+$; Found: 354.1710.

6-(1H-indol-1-yl)-9-propyl-2-(4-(trifluoromethyl)phenyl)-9H-purine (**AIP-CF**)
AIP-CF was produced using the same procedure as **AIP** by changing the phenylboronic acid as (4-(Trifluoromethyl)phenyl)boronic acid. White solid in 91% yield. ^{1}H NMR (400 MHz, d^{6}-DMSO): δ (TMS, ppm) 9.14 (d, J = 3.6 Hz, 1H), 8.92 (d, J = 8.3 Hz, 1H), 8.60 (s, 1H), 8.55 (d, J = 8.1 Hz, 2H), 7.87 (d, J = 8.2 Hz, 2H), 7.66 (d, J = 7.7 Hz, 1H),

7.37 (t, J = 7.7 Hz, 1H), 7.26 (t, J = 7.4 Hz, 1H), 6.87 (d, J = 3.6 Hz, 1H), 4.22 (t, J = 7.1 Hz, 2H), 1.95 – 1.86 (m, 2H), 0.87 (t, J = 7.4 Hz, 3H).^{13}C NMR (100 MHz, d^{6}-DMSO): δ (TMS, ppm) 156.28, 153.90, 149.01, 146.29, 141.81, 135.61, 130.62, 128.88, 126.24, 126.20, 124.42, 123.25, 121.46, 121.42, 116.74, 108.64, 45.48, 22.99, 11.47. HRMS (ESI): *m/z*: Calcd for $C_{22}H_{19}F_3N_5^+$: 422.1592 [*M*+*H*]$^+$; Found: 422.1600.

4-(6-(1H-indol-1-yl)-9-propyl-9H-purin-2-yl)benzonitrile (**AIP-CN**)

AIP-CN was produced using the same procedure as **AIP** by changing the phenylboronic acid as 4-cyanophenylboronicacid. White solid in 91% yield. ^{1}H NMR (400 MHz, d^{6}-DMSO): δ (TMS, ppm) 9.19 (d, J = 3.7 Hz, 1H), 8.95 (d, J = 8.3 Hz, 1H), 8.69 (s, 1H), 8.60 (d, J = 8.4 Hz, 2H), 8.04 (d, J = 8.4 Hz, 2H), 7.69 (d, J = 7.7 Hz, 1H), 7.41 (t, J = 7.3 Hz, 1H), 7.29 (t, J = 7.3 Hz, 1H), 6.92 (d, J = 3.6 Hz, 1H), 1.94 (h, J = 7.3 Hz, 2H), 0.90 (t, J = 7.4 Hz, 3H).^{13}C NMR (100 MHz, $CDCl_3$): δ (TMS, ppm) 156.72, 153.60, 149.68, 143.32, 142.37, 135.84, 132.35, 130.76, 128.78, 128.64, 123.90, 122.90, 121.74, 121.00, 118.92, 116.50, 113.32, 108.52, 45.70, 23.31, 11.31. HRMS (ESI): *m/z*: Calcd for $C_{23}H_{19}N_6^+$:379.1671 [*M*+*H*]$^+$; Found: 379.1671.

4-(6-(1H-indol-1-yl)-9-propyl-9H-purin-2-yl)benzaldehyde (**AIP-CHO**)

AIP-CHO was produced using the same procedure as **AIP** by changing the phenylboronic acid as 4-formylphenylboronic acid. White solid in 88% yield. ^{1}H NMR (400 MHz, d^{6}-DMSO): δ (TMS, ppm) 10.10 (s, 1H), 9.20 (d, J = 3.7 Hz, 1H), 9.01 (d, J = 8.3 Hz, 1H), 8.71 – 8.65 (m, 3H), 8.11 (d, J = 8.3 Hz, 2H), 7.70 (d, J = 7.7 Hz, 1H), 7.44 (t, J = 7.3 Hz, 1H), 7.29 (t, J = 7.2 Hz, 1H), 6.93 (d, J = 3.6 Hz, 1H), 4.33 (t, J = 7.0 Hz, 2H), 1.96 (h, J = 7.2 Hz, 2H), 0.91 (t, J = 7.4 Hz, 3H).^{13}C NMR (100 MHz, d^{6}-DMSO): δ (TMS, ppm) 193.38, 156.59, 153.98, 149.03, 146.43, 143.29, 137.58, 135.63, 130.63, 130.45, 128.92, 128.83, 124.48, 123.28, 121.51, 121.45, 116.74, 108.68, 45.51, 23.03, 11.49. HRMS (ESI): *m/z*: Calcd for $C_{23}H_{20}N_5O_1^+$: 382.1668 [*M*+*H*]$^+$; Found: 382.1653.

2-(4-(6-(1H-indol-1-yl)-9-propyl-9H-purin-2-yl)benzylidene)malononitrile (**AIP-CN2**)

After **AIP-CHO** (381 mg, 1 mmol) and malononitrile (110 mg, 1.3 mmol) was added in DMF (5 mL), the piperidine (0.05 mL) was dropped to the stirred solution. Then the mixture was heated at reflux for about 2 h. After the reaction was completed based on the TLC, poured the reaction mixture into water and extracted with DCM. The organic layer was washed with brine, water and dried over anhydrous Na_2SO_4. The crude product was purified by column chromatography on silica gel. Elution with DCM gave **AIP-CN2** as a yellow solid in 90% yield. ^{1}H NMR (400 MHz, d^{6}-DMSO): δ (TMS, ppm) 9.18 (d, J = 3.7 Hz, 1H), 8.94 (d, J = 8.5 Hz, 1H), 8.66 (s, 1H), 8.60 – 8.53 (m, 3H), 8.08 (d, J = 8.5 Hz, 2H), 7.68 (d, J = 7.6 Hz, 1H), 7.43 (t, J = 7.2 Hz, 1H), 7.28 (t, J = 7.1 Hz, 1H), 6.91 (d, J = 3.7 Hz, 1H), 4.27 (t, J = 7.1 Hz, 2H), 1.92 (q, J = 7.3 Hz, 2H), 0.89 (t, J = 7.4 Hz, 3H). ^{13}C NMR (101 MHz,$CDCl_3$) δ 159.17, 156.59, 153.56,

149.67, 143.90, 143.44, 135.83, 131.97, 131.03, 130.75, 129.20, 128.65, 124.01, 122.96, 121.84, 121.02, 116.54, 113.81, 112.70, 108.58, 82.80, 45.74, 23.32, 11.32. HRMS (ESI): *m/z*: Calcd for $C_{26}H_{20}N_7^+$: 430.1780 $[M+H]^+$; Found: 430.1777.

1.2 Cell culture

Hela cells were cultured in Dulbecco's modified Eagle medium (DMEM) containing 10% fetal bovine serum and 1% Antibiotic–antimycotic at 37℃ in a 5% CO_2/95% air incubator. For fluorescence imaging, cells (4 × 10^3/well) were passed on a 6-well plate and incubated for 24 h.

1.2 Cytotoxicity study

Toxicity toward HeLa cells was determined by MTS (3-(4,5-dimethylthiazol-2-yl)-5-(3-carboxymethoxyphenyl)-2-(4-sulfophenyl)-2H-tetrazolium) reduction assay following literature procedures. About 10000 cells per well were seeded in 96-well plates and cultured overnight for 70-80% cell confluence. The medium was replaced with 100 μL of fresh medium with different concentration of probes, to which 100 μL complexes at 200 μL. 24 hours later, 100 μL of 20% MTT solution in PBS was replaced with the old medium in each well for additional 0.5h incubation. The metabolic activity of the probes treated cells was expressed as a relative to untreated cell controls taken as 100% metabolic activity.

1.3 Cell imaging

HeLa cells were grown on a cover slip overnight in a 35-mm petri dish. The cells were stained with certain dye at certain concentration for certain time (by adding 2 μL of stock solution in DMSO to a 2 mL of culture medium with DMSO < 0.1 vol %). The cells were imaged under a fluorescent microscope (upright BX41 Microscope) using proper excitation and emission filters for each dye: for probes, λ_{ex}= 405 nm, λ_{em}= 420-480 nm; for BODIPY 493/503, λ_{ex}= 488 nm, λ_{em}= 530-560 nm.

2. Molar Extinction Coefficient of all compounds

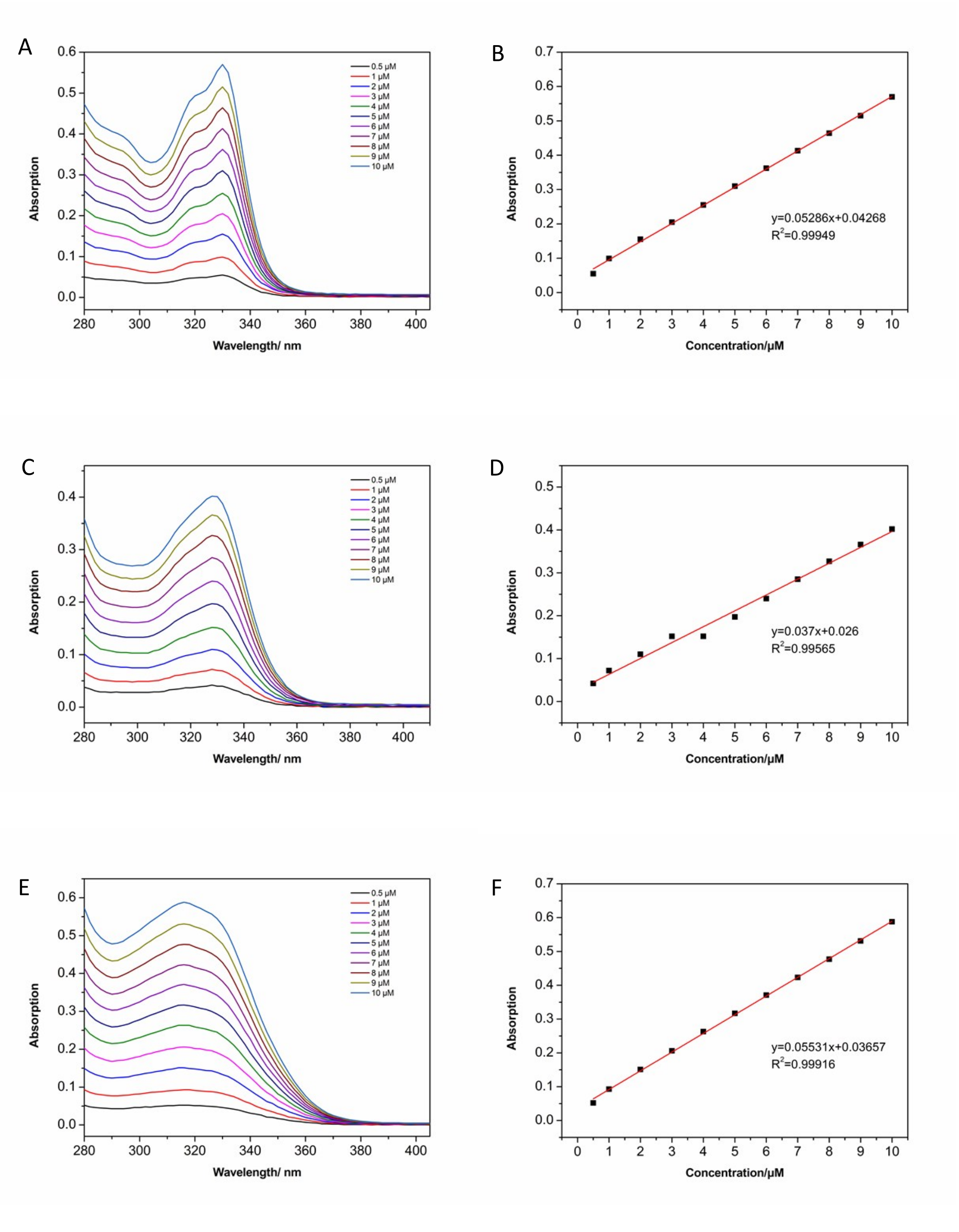

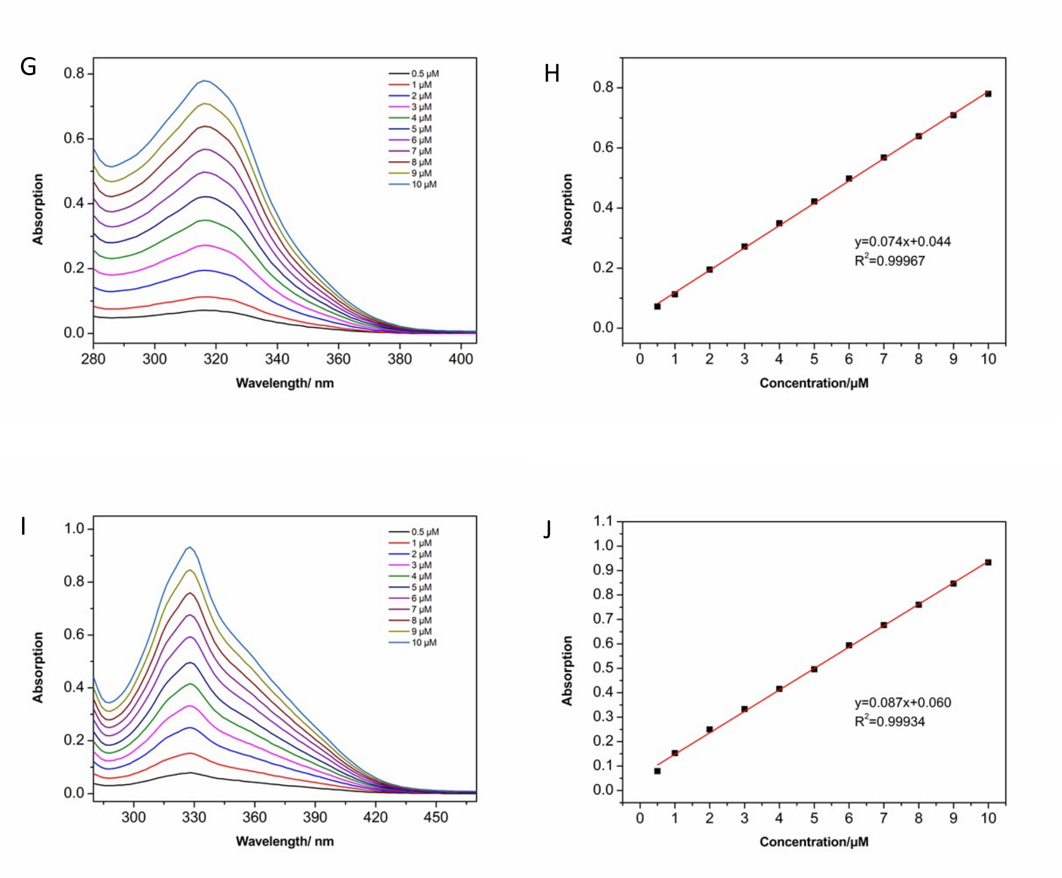

Fig. S1. UV spectra of compound A) AIP, C) AIP-CF, E) AIP-CN, G) AIP-CHO, I) AIP-CN2 at different concentrations (0.5, 1, 2, 3, 5, 6, 8, 10 μM); Absorption-concentration curve of compound B) AIP, D) AIP-CF, F) AIP-CN, H) AIP-CHO, J) AIP-CN2.

Table S1. Summary of all the compounds' molar extinction coefficient

Compounds	λ_{Abs} (nm)	ε(M^{-1} cm^{-1})
A-In-Ph	330	5.70×10^5
A-In-CF	328	4.02×10^5
A-In-CN	316	5.88×10^5
A-In-CHO	316	7.80×10^5
A-In-CN2	328	9.33×10^5

3. Solvent effect of all compounds

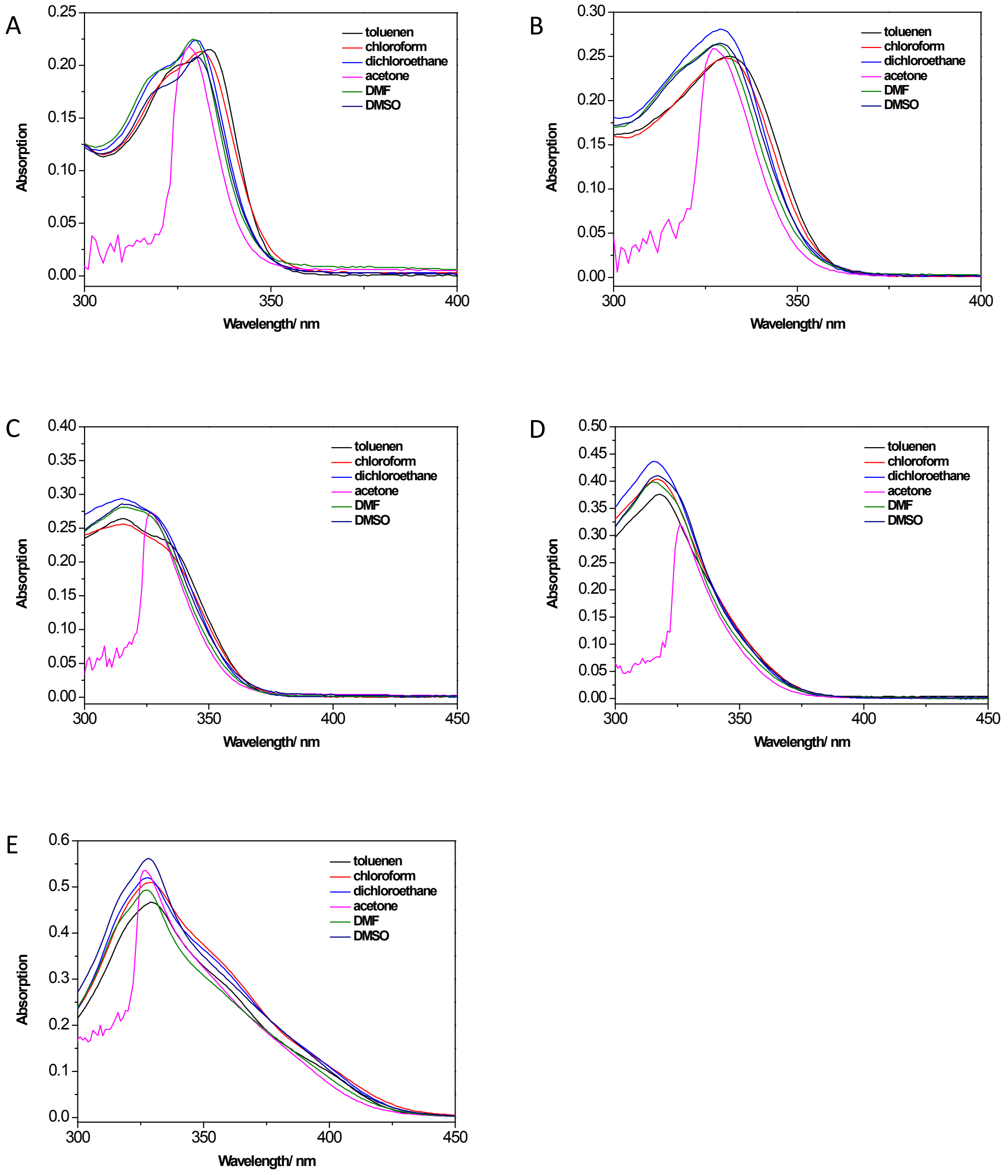

Fig. S2. Absorption spectra of A) AIP, B) AIP-CF, C) AIP-CN, D) AIP-CHO, E) AIP-CN2 in different solvents.

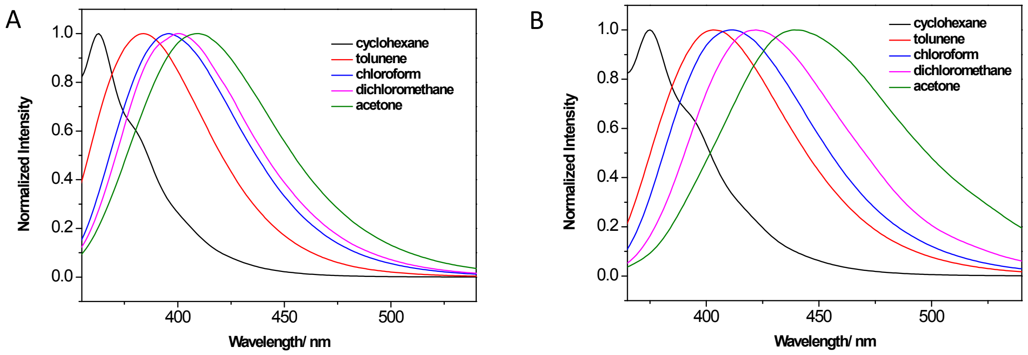

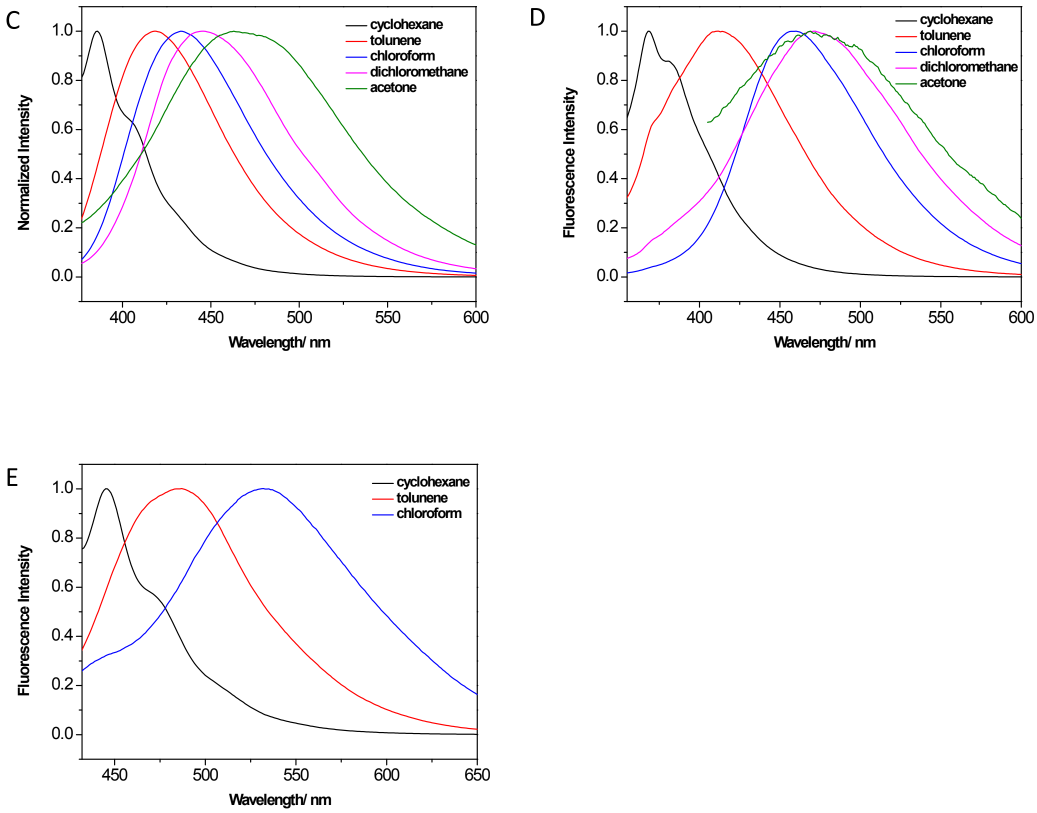

Fig. S3. Normalized fluorescence spectra of A) AIP, B) AIP-CF, C) AIP-CN, D) AIP-CHO, E) AIP-CN2 in different solvents.

Table S2. Optical transitions of all the compounds in different solvents

	cyclohexane			toluene		chlororform		dichloromethane		acetone	
Compound	λ_{ab} (nm)	λ_{em} (nm)	Stokes shift (nm)	λ_{em} (nm)	Stokes shift (nm)	λ_{em} (nm)	Stokes shift (nm)	λ_{em} (nm)	Stoke s shift (nm)	λ_{em} (nm)	Stokes shift (nm)
A-In-Ph	331	363	32	384	53	395	64	400	69	409	78
A-In-CF	336	375	39	404	68	411	75	421	85	440	104
A-In-CN	337	385	48	418	81	432	95	447	110	467	130
A-In-CHO	338	383	45	412	74	459	121	471	133	478	140
A-In-CN2	362	446	84	485	123	533	171	——	——	——	——

4. Fluorescence spectra of all compounds in DMSO/PBS mixtures

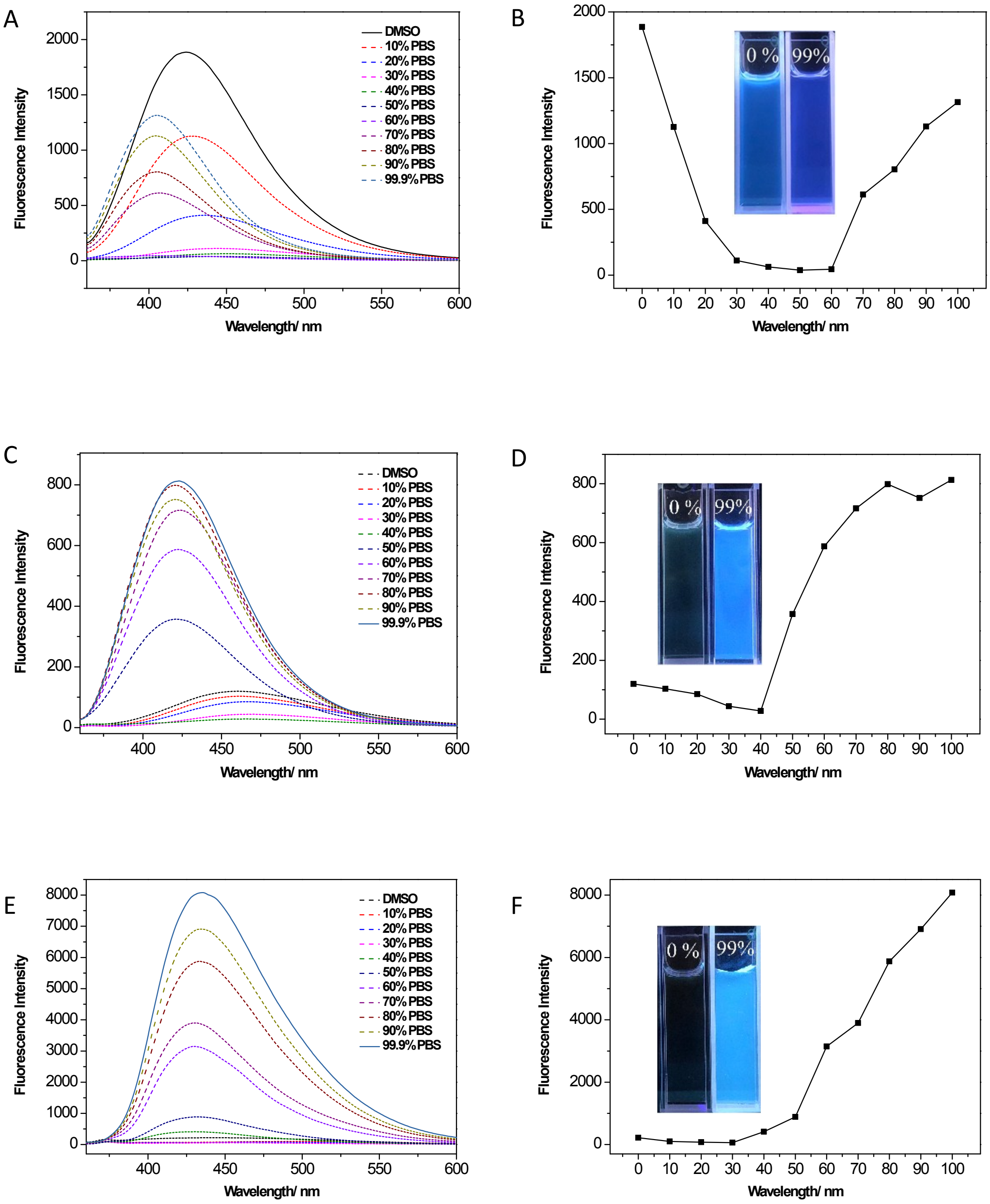

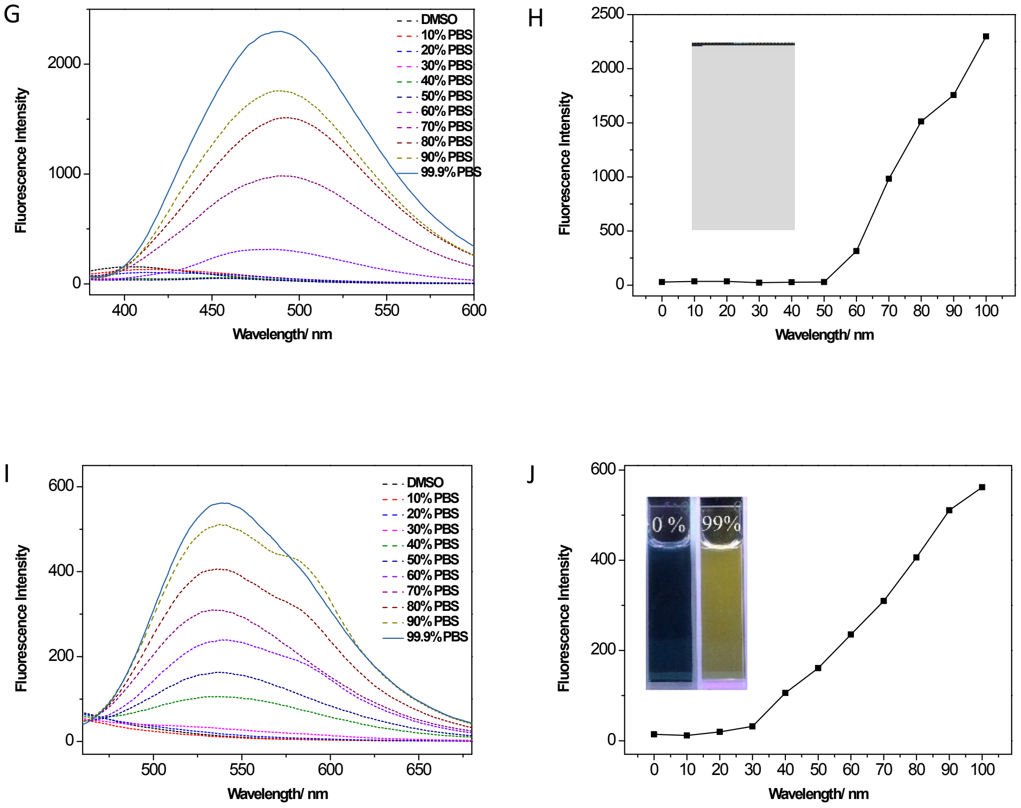

Fig. S4. Fluorescence spectra of all the compounds in DMSO/PBS mixtures and dependence of the I/I_0 ratios of all the compounds on the solvent composition of the DMSO/PBS mixture. A) and B): AIP; C) and D): AIP-CF; E) and F): AIP-CN; G) and H): AIP-CHO; I) and J): AIP-CN2; Concentration: 5 μM, λ_{ex} = 340 nm. Insert: photographs of each compound in DMSO/PBS mixtures with f_P values of 0 and 99% under irradiation with 365 nm UV light.

5. Particle size of all compounds in aggregation state

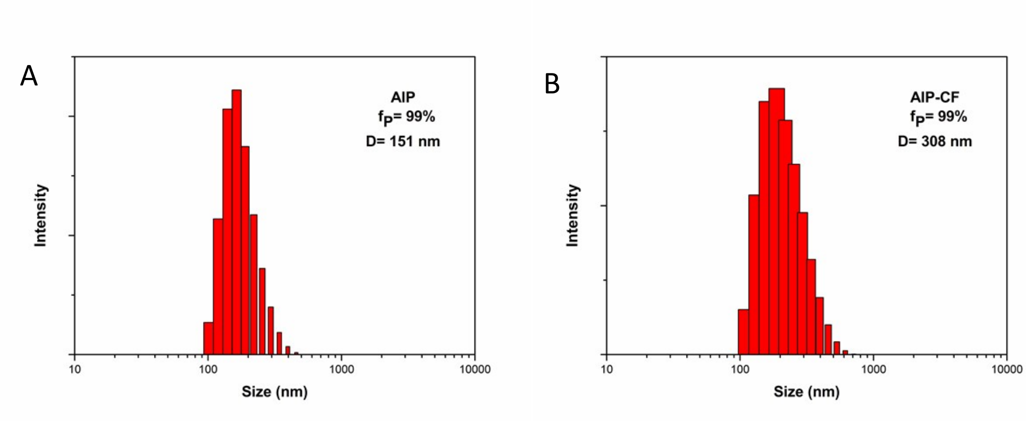

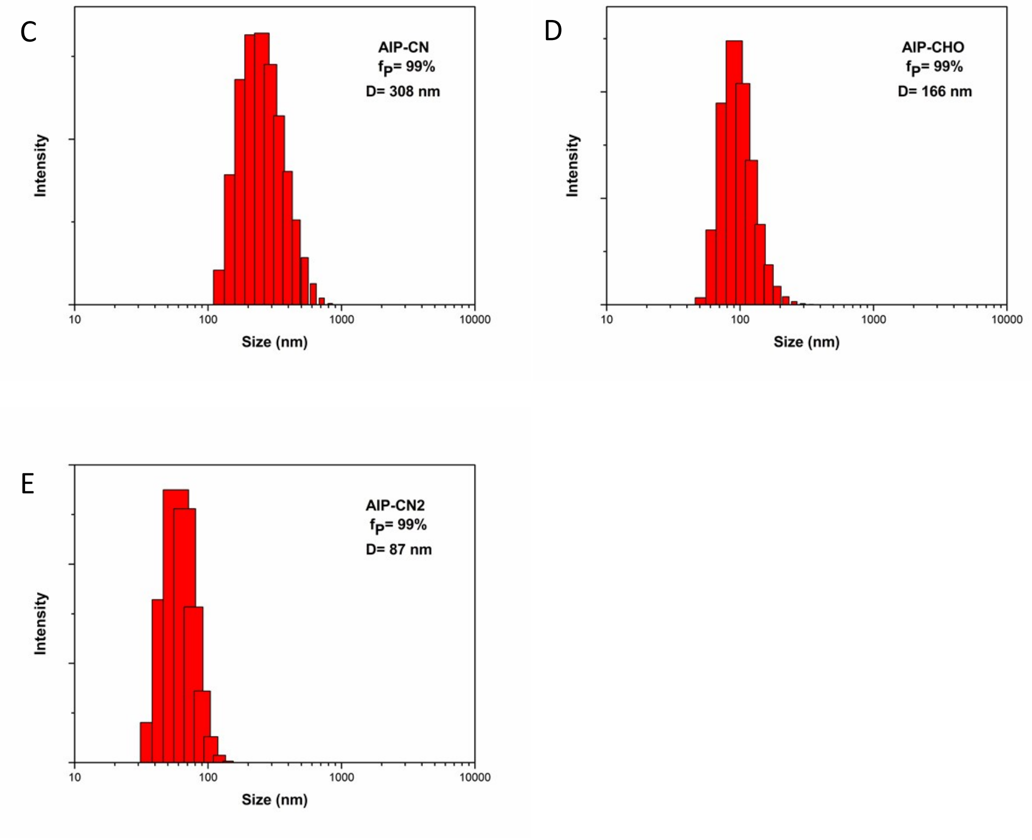

Fig. S5 Particle size distribution of all compounds (10 μM) in DMSO/PBS mixture with a f_P value of 99%.

6. Solid fluorescence spectra of all compounds and their CIE diagram

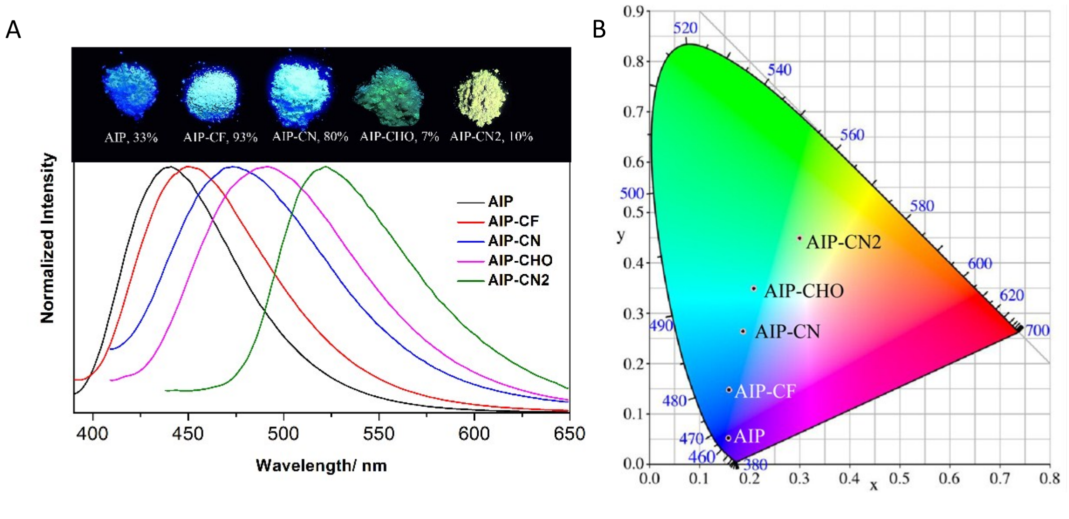

Fig. S6 A)Solid fluorescence spectra of all the compounds, λ_{ex} = 370 nm; B) fluorescence spectra of all compounds plotted on a CIE 1931 chromaticity diagram.

Table S3. Coordinates of compounds 1a-1d and 2a-2g on CIE diagram.

Compound	λ_{em}(nm)	Coordinate (X)	Coordinate (Y)
AIP	407	0.1574	0.0515
AIP-CF	450	0.1589	0.1474
AIP-CN	473	0.1865	0.2635
AIP-CHO	490	0.2084	0.3487
AIP-CN2	522	0.3004	0.4490

7. Fluorescent lifetime and quantum yield of all compounds in solution, aggregation and in solid

Table S4. Fluorescent lifetime of all the compounds in DMSO, PBS and in solid state.

Compd.	Lifetime in soluetion (s)	Lifetime in aggregation (s)	Lifetime in solid state (s)
AIP	τ_1=9.60×10^{-10} (59%)<br>τ_2=8.29×10^{-9} (41%)<br>τ_{avg}=3.96×10^{-9}	τ_1=1.41×10^{-10} (64%)<br>τ_2=4.55×10^{-9} (36%)<br>τ_{avg}=1.73×10^{-9}	τ_1=2.39×10^{-11} (85%)<br>τ_2=3.49×10^{-9} (15%)<br>τ_{avg}=5.44×10^{-10}
AIP-CF	τ_1=3.13×10^{-9} (38%)<br>τ_2=8.30×10^{-10} (52%)<br>τ_3=1.10×10^{-8} (10%)<br>τ_{avg}=2.72×10^{-9}	τ_1=6.56×10^{-11} (33%)<br>τ_2=4.20×10^{-11} (44%)<br>τ_3=6.37×10^{-12} (23%)<br>τ_{avg}=4.16×10^{-11}	τ_1=1.74×10^{-9} (27%)<br>τ_2=3.41×10^{-11} (30%)<br>τ_3=8.44×10^{-9} (43%)<br>τ_{avg}=4.11×10^{-9}
AIP-CN	τ_1=3.43×10^{-9} (22%)<br>τ_2=8.52×10^{-10} (66%)<br>τ_3=1.18×10^{-8} (12%)<br>τ_{avg}=2.73×10^{-9}	τ_1=4.96×10^{-9} (33%)<br>τ_2=1.45×10^{-8} (54%)<br>τ_3=3.00×10^{-10} (13%)<br>τ_{avg}=9.51×10^{-9}	τ_1=5.78×10^{-9} (39%)<br>τ_2=9.96×10^{-9} (61%)<br>τ_{avg}=8.33×10^{-9}
AIP-CHO	τ_1=3.78×10^{-9} (19%)<br>τ_2=6.21×10^{-10} (71%)<br>τ_3=2.98×10^{-8} (10%)<br>τ_{avg}=4.14×10^{-9}	τ_1=6.03×10^{-9} (47%)<br>τ_2=1.92×10^{-8} (40%)<br>τ_3=1.56×10^{-10} (13%)<br>τ_{avg}=1.05×10^{-8}	τ_1=2.45×10^{-2} (58%)<br>τ_2=2.74×10^{-3} (42%)<br>τ_{avg}=1.53×10^{-2}
AIP-CN2	τ_1=2.81×10^{-9} (27%)<br>τ_2=3.13×10^{-10} (59%)<br>τ_3=1.11×10^{-8} (14%)<br>τ_{avg}=2.50×10^{-9}	τ_1=2.70×10^{-9} (8%)<br>τ_2=1.12×10^{-8} (11%)<br>τ_3=4.86×10^{-11} (81%)<br>τ_{avg}=3.78×10^{-9}	τ_1=1.06×10^{-2} (41%)<br>τ_2=2.00×10^{-3} (59%)<br>τ_{avg}=5.53×10^{-3}
AIP-Py	τ_1=3.35×10^{-9} (48%)<br>τ_2=5.92×10^{-10} (33%)<br>τ_3=1.22×10^{-8} (19%)<br>τ_{avg}=4.12×10^{-9}	τ_1=4.54×10^{-9} (40%)<br>τ_2=9.58×10^{-10} (18%)<br>τ_3=1.26×10^{-8} (42%)<br>τ_{avg}=7.28×10^{-9}	τ_1=1.43×10^{-1} (42%)<br>τ_2=1.29×10^{-2} (58%)<br>τ_{avg}=6.76×10^{-2}

Table S5. Quantum yield of all the compounds in DMSO, PBS and in solid state.

Compd.	Quantum yield in solution (%)	Quantum yield in aggregation (%)	Quantum yield in solid state (%)
AIP	87.6	23.6	33.0
AIP-CF	10.9	26.4	93.5
AIP-CN	2.2	37.9	80.2
AIP-CHO	1.3	5.1	7.3
AIP-CN2	0.7	5.0	10.2

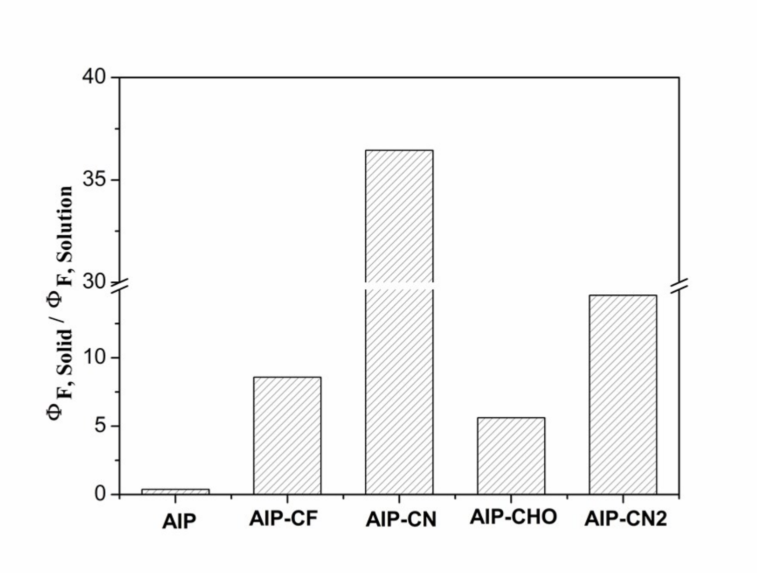

Fig. S7 The ratio of the quantum yields for the solid and solution states of all the compounds.

Table S6. The rate constants for radiative (k_r) and nonradiative decay (k_{nr}) were calculated from the Φ and τ values according to the formulae $k_r = \Phi_F/\tau$ and $k_{nr} = (1-\Phi_F)/\tau$.

Compound	Solution		aggregation		Solid state	
	K_r (s^{-1})	K_{nr} (s^{-1})	K_r (s^{-1})	K_{nr} (s^{-1})	K_r (s^{-1})	K_{nr} (s^{-1})
AIP	2.21×10^8	3.13×10^7	1.36×10^8	4.24×10^8	6.07×10^8	1.23×10^9
AIP-CF	4.01×10^7	3.28×10^8	6.35×10^9	1.77×10^{10}	2.27×10^8	1.58×10^7
AIP-CN	8.06×10^6	3.58×10^8	3.99×10^7	6.53×10^7	9.63×10^7	2.38×10^7
AIP-CHO	3.14×10^6	2.38×10^8	4.86×10^6	9.04×10^7	4.77	6.06×10^2
AIP-CN2	2.80×10^6	3.97×10^8	1.32×10^7	2.51×10^8	1.84×10^2	1.62×10^3

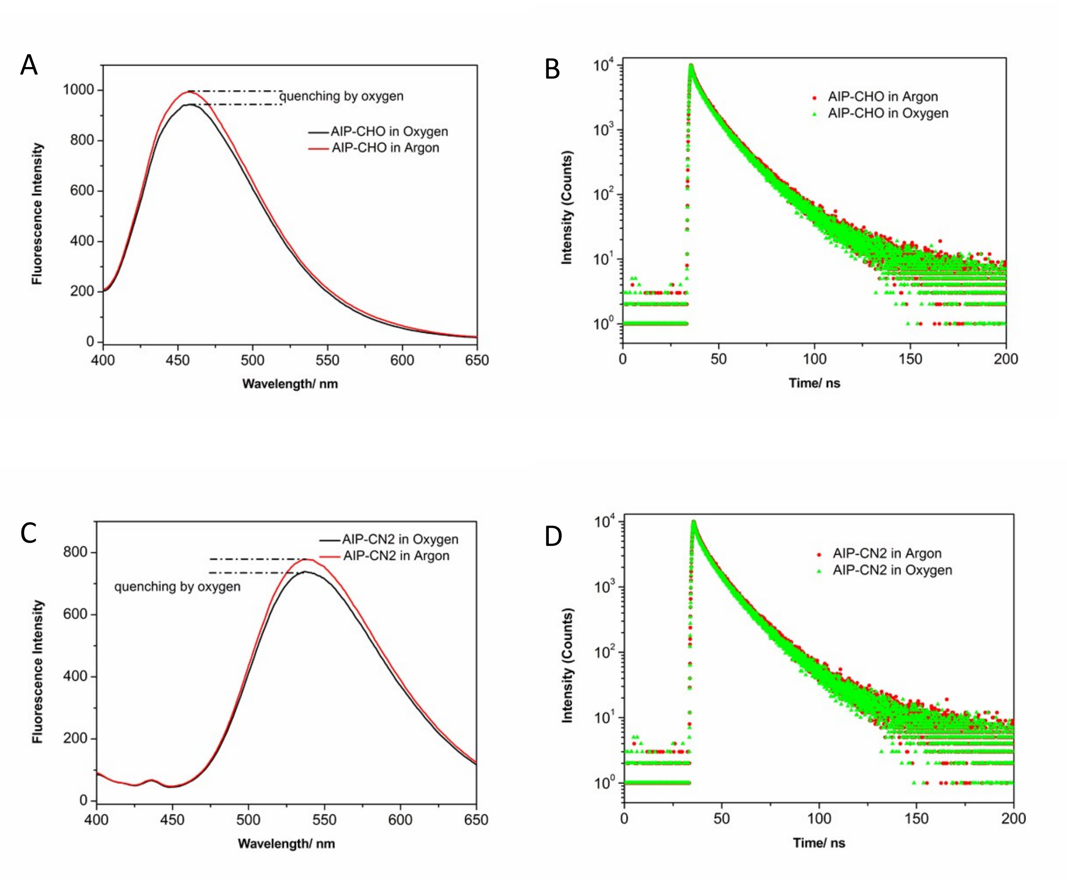

Fig. S8 Fluorescence spectra and emission decay of A,B) AIP-CHO, C,D) AIP-CN2 with argon/oxygen in PBS solution.

8. Theoretical Calculation of all compounds

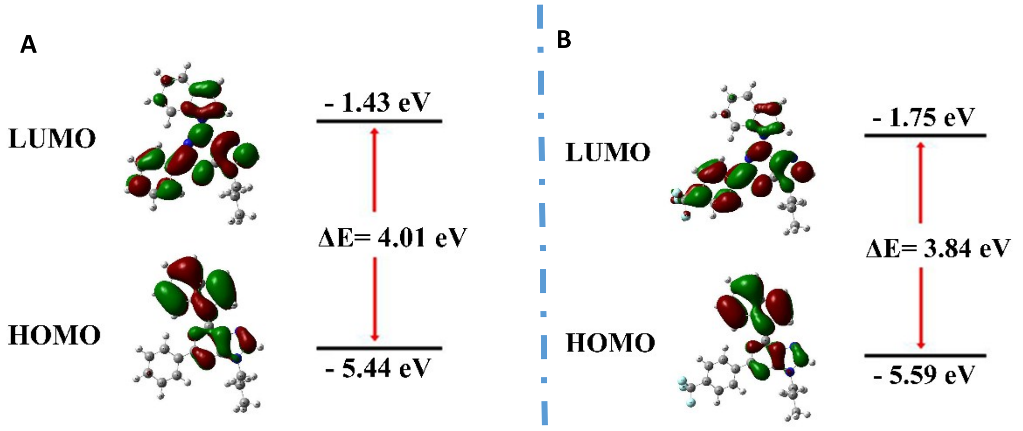

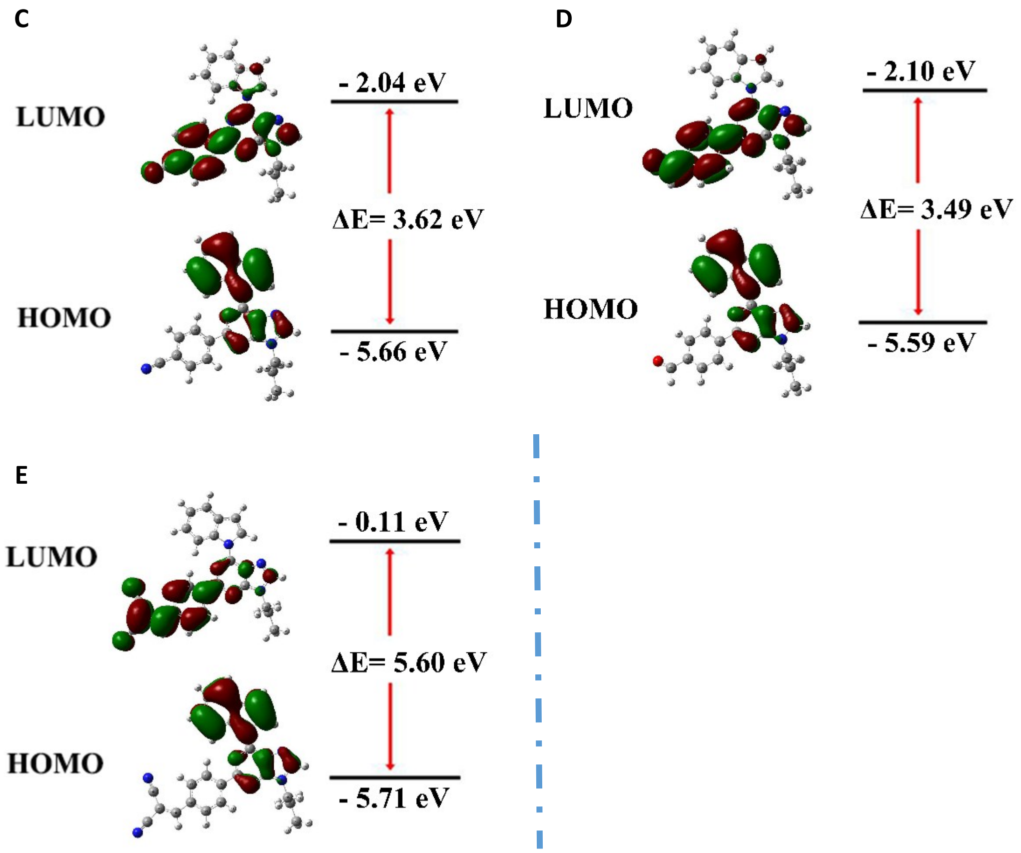

Fig. S9 Molecular orbital amplitude plots of HOMO and LUMO levels of A) AIP, B) AIP-CF, C) AIP-CN, D) AIP-CHO and E) AIP-CN2 calculated at the B3LYP/6-31G (d, p) level of theory.

9. Views of the molecular stacking structures in single crystals of AIP, AIP-CF, AIP-CN and AIP-CHO

A
B

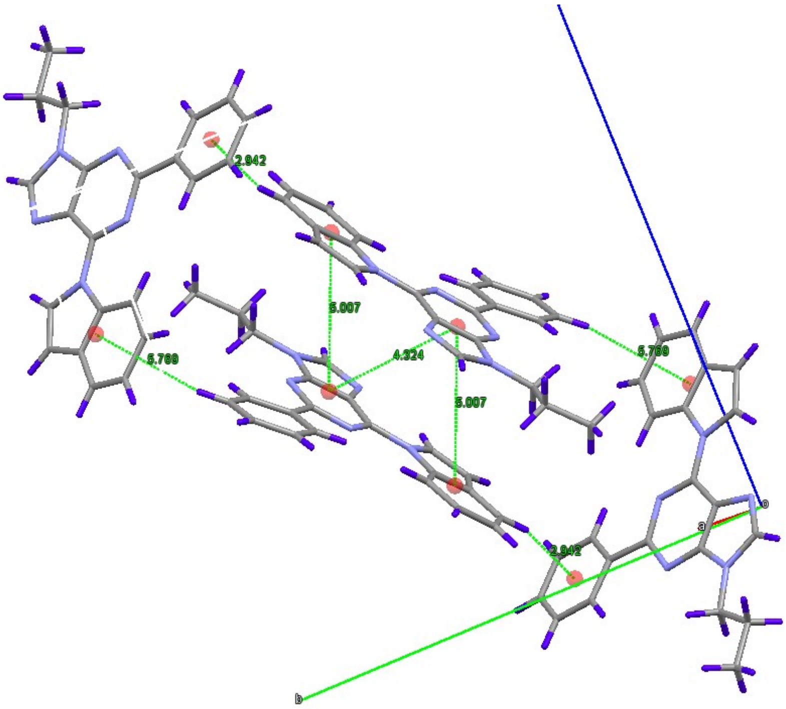
2.942
5.007
4.324
5.769
5.007
5.769
2.942
a
b

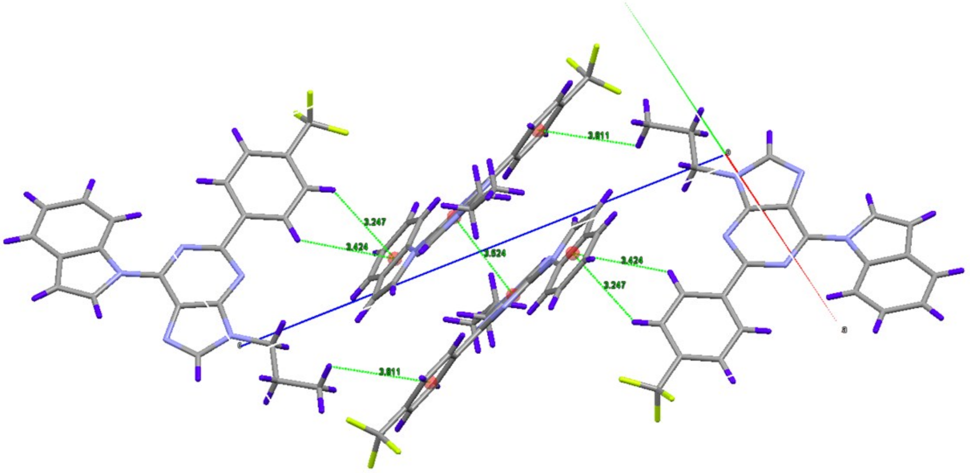
3.811
3.247
3.424
3.524
3.424
3.247
3.811

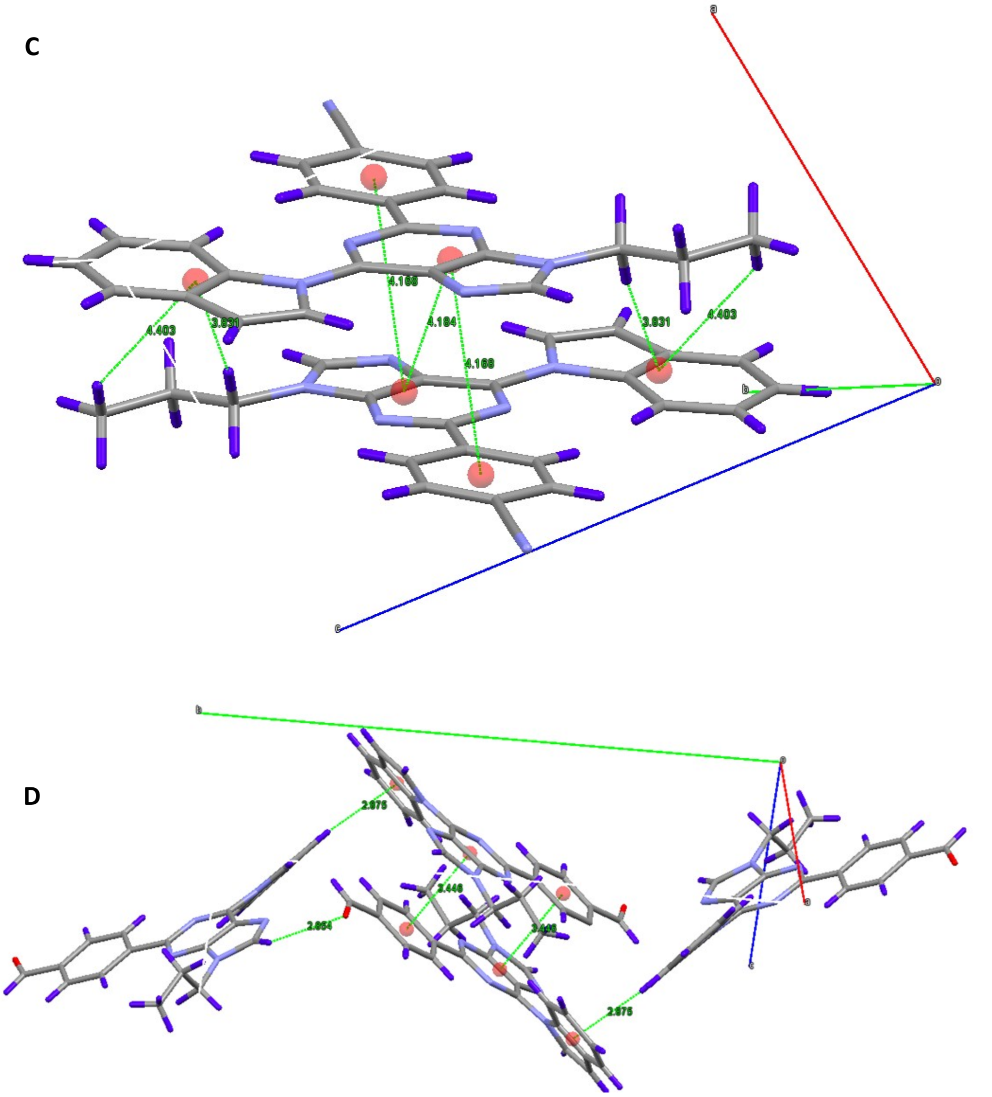

Fig. S10 Side and top view of crystal packing mode of A) AIP, B) AIP-CF, C) AIP-CN and D) AIP-CHO. Carbon, hydrogen, oxygen and nitrogen atoms are shown in gray, blue, red and mauve, respectively.

Table S6. The dihedral angle and the distance data of AIP, AIP-CF, AIP-CN, and AIP-CHO.

Compounds	$Ø_{P\text{-}D}{}^{a}$	$Ø_{P\text{-}A}{}^{a}$	$d_{D\text{-}A}{}^{b}$	$d_{D\text{-}P}{}^{b}$	$d_{A\text{-}P}{}^{b}$	$d_{P\text{-}P}{}^{b}$	μ^{c}
AIP	4.91	11.96	8.439	5.007	7.927	4.324	4.6118
AIP-CF	5.63	5.76	7.423	5.347	6.067	3.524	6.0123
AIP-CN	12.38	11.09	6.361	6.21	4.168	4.184	7.7259
AIP-CHO	24.64	5.34	5.577	7.262	3.446	5.993	7.4134

[a]The dihedral angle of purine core and donor group ($Ø_{P\text{-}D}$) or accepter group ($Ø_{P\text{-}A}$). [b]The distance of adjacent molecule's purine core (P), donor group (D), and accepter group (A). [c]Dipole moment of each molecule calculated at the B3LYP/6-31G (d, p) level of theory based on the single crystal.

10. Crystallographic data of AIP, AIP-CF, AIP-CN and AIP-CHO

Crystal data and structure refinements of AIP:

Identification code	AIP
Empirical formula	$C_{22}H_{19}N_5$
Formula weight	353.42
Temperature/K	295.5(5)
Crystal system	monoclinic
Space group	$P2_1/n$
a/Å	6.8768(3)
b/Å	15.8540(8)
c/Å	16.6138(6)
α/°	90
β/°	91.265(4)
γ/°	90
Volume/Å^3	1810.87(13)
Z	4
ρ_{calc}g/cm^3	1.296
μ/mm^{-1}	0.631
F(000)	744.0
Crystal size/mm^3	0.7 × 0.4 × 0.2
Radiation	CuKα (λ = 1.54184)
2Θ range for data collection/°	7.708 to 145.774
Index ranges	$-8 \leq h \leq 7$, $-19 \leq k \leq 16$, $-20 \leq l \leq 13$
Reflections collected	10173
Independent reflections	3557 [R_{int} = 0.0340, R_{sigma} = 0.0287]
Data/restraints/parameters	3557/0/245
Goodness-of-fit on F^2	1.040
Final R indexes [I>=2σ (I)]	R_1 = 0.0617, wR_2 = 0.1606
Final R indexes [all data]	R_1 = 0.0697, wR_2 = 0.1738
Largest diff. peak/hole / e Å^{-3}	0.19/-0.36
CCDC number	1855630

Crystal data and structure refinements of AIP-CF:

Identification code	AIP-CF
Empirical formula	$C_{23}H_{18}F_3N_5$
Formula weight	421.42
Temperature/K	296.5(6)
Crystal system	monoclinic

Space group	$P2_1/c$
a/Å	13.8657(4)
b/Å	8.21008(19)
c/Å	18.9838(6)
α/°	90
β/°	111.052(3)
γ/°	90
Volume/Å^3	2016.84(10)
Z	4
ρ_{calc}g/cm^3	1.388
μ/mm^{-1}	0.880
F(000)	872.0
Crystal size/mm^3	0.7 × 0.65 × 0.6
Radiation	CuKα (λ = 1.54184)
2Θ range for data collection/°	9.868 to 134.142
Index ranges	$-16 \leq h \leq 11$, $-9 \leq k \leq 9$, $-22 \leq l \leq 22$
Reflections collected	13884
Independent reflections	3599 [R_{int} = 0.0215, R_{sigma} = 0.0152]
Data/restraints/parameters	3599/6/311
Goodness-of-fit on F^2	1.086
Final R indexes [I>=2σ (I)]	R_1 = 0.0657, wR_2 = 0.1531
Final R indexes [all data]	R_1 = 0.0700, wR_2 = 0.1593
Largest diff. peak/hole / e Å^{-3}	0.39/-0.51
CCDC number	1855631

Crystal data and structure refinements of AIP-CN:

Identification code	AIP-CN
Empirical formula	$C_{23}H_{18}N_6$
Formula weight	378.43
Temperature/K	296.3(5)
Crystal system	triclinic
Space group	P-1
a/Å	8.0380(6)
b/Å	10.3402(8)
c/Å	11.9954(9)
α/°	73.536(7)
β/°	81.046(7)
γ/°	84.939(6)
Volume/Å^3	943.43(13)

Z	2
ρ_{calc}g/cm^3	1.332
μ/mm^{-1}	0.661
F(000)	396.0
Crystal size/mm^3	0.65 × 0.4 × 0.3
Radiation	CuKα (λ = 1.54184)
2Θ range for data collection/°	7.758 to 146.426
Index ranges	-6 ≤ h ≤ 9, -12 ≤ k ≤ 12, -14 ≤ l ≤ 14
Reflections collected	10665
Independent reflections	3698 [R_{int} = 0.0264, R_{sigma} = 0.0231]
Data/restraints/parameters	3698/0/263
Goodness-of-fit on F^2	1.047
Final R indexes [I>=2σ (I)]	R_1 = 0.0684, wR_2 = 0.1844
Final R indexes [all data]	R_1 = 0.0782, wR_2 = 0.1999
Largest diff. peak/hole / e Å^{-3}	0.53/-0.34
CCDC number	1855632

Crystal data and structure refinements of AIP-CHO:

Identification code	AIP-CHO
Empirical formula	$C_{23}H_{19}N_5O$
Formula weight	380.42
Temperature/K	297.5(3)
Crystal system	monoclinic
Space group	P2$_1$/c
a/Å	11.7851(4)
b/Å	19.9797(6)
c/Å	8.1892(3)
α/°	90
β/°	97.235(3)
γ/°	90
Volume/Å^3	1912.90(10)
Z	4
ρ_{calc}g/cm^3	1.321
μ/mm^{-1}	0.679
F(000)	796.0
Crystal size/mm^3	0.6 × 0.4 × 0.3
Radiation	CuKα (λ = 1.54184)
2Θ range for data collection/°	7.562 to 145.42
Index ranges	-14 ≤ h ≤ 14, -22 ≤ k ≤ 24, -6 ≤ l

	≤ 9
Reflections collected	10922
Independent reflections	3743 [R_{int} = 0.0294, R_{sigma} = 0.0259]
Data/restraints/parameters	3743/6/304
Goodness-of-fit on F^2	1.019
Final R indexes [I>=2σ (I)]	R_1 = 0.0588, wR_2 = 0.1624
Final R indexes [all data]	R_1 = 0.0679, wR_2 = 0.1753
Largest diff. peak/hole / e Å^{-3}	0.29/-0.30
CCDC number	1855634

11. Cytotoxicity of all probes on Hela cells evaluated by MTS assay

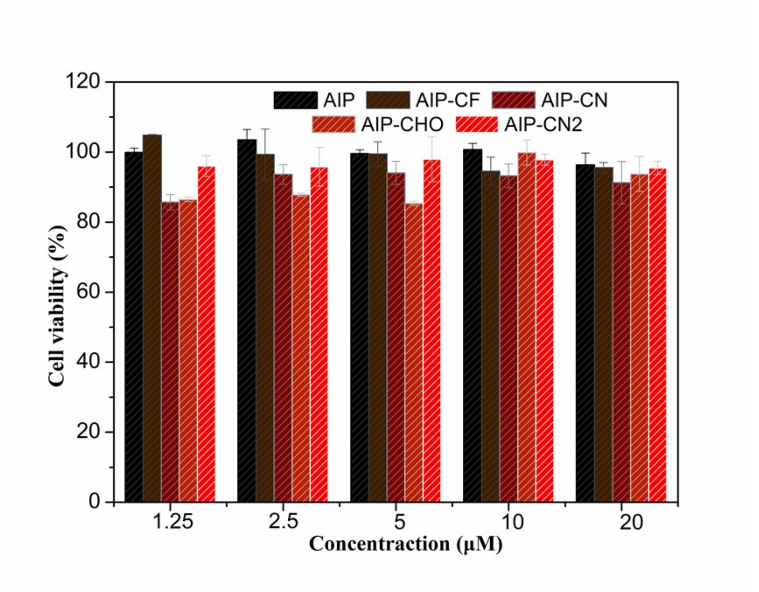

Fig. S11 Cell viabilities of Hela cells after incubation with different concentrations of all probes. (1.25, 2.5, 5, 10, 20 μM) for 24 h.

12. Single-photo CLSM images of Hela cells incubated with all probes and BODIPY 493/503

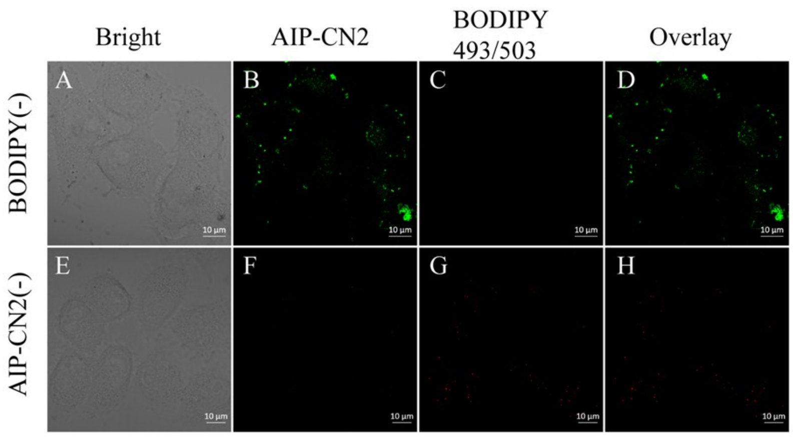

Fig. S12 Live Hela cells incubated with (A-D) AIP-CN2, (E-H) BODIPY 493/503. Channel of **AIP-CN2**, λ_{em}=420-480, channel of BODIPY 493/503, λ_{em}=530-560 nm.

13. Photostability of all probes in CLSM imaging

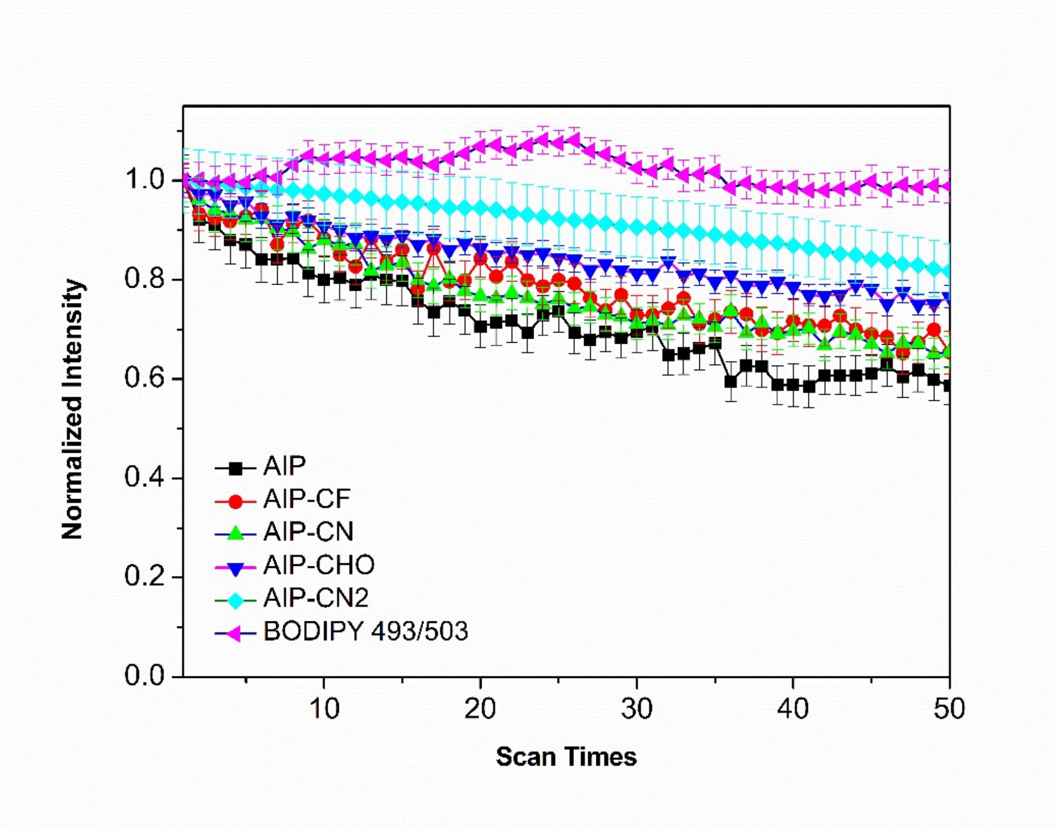

Fig. S13 Fluorescent signal change of HeLa cells stained with AIEgens and BODIPY 493/503 with the number of scans of laser irradiation. Concentration: 1 μM; dyeing time: 30 min; excitation wavelength: 405 nm; emission wavelength: 420-600 nm; laser power: 0.3 μW; scanning rate: 1.6 s per time.

14. NMR Data

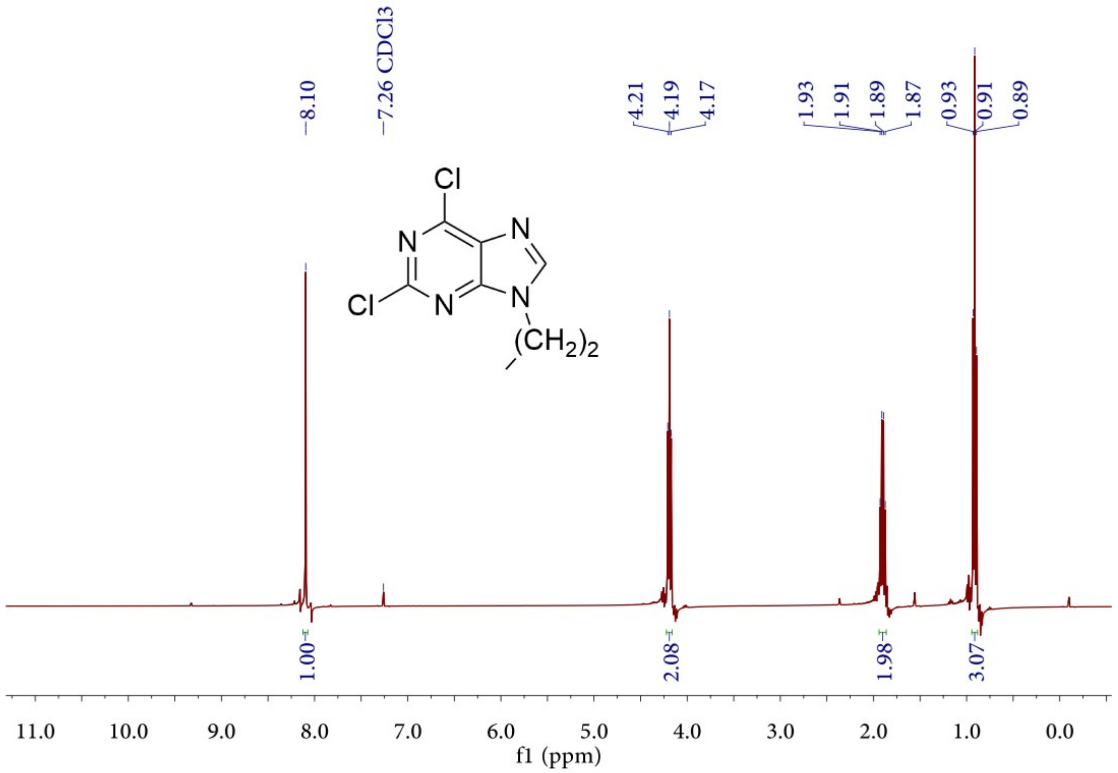

^{1}H NMR of Compound **2** in d^{6}-DMSO

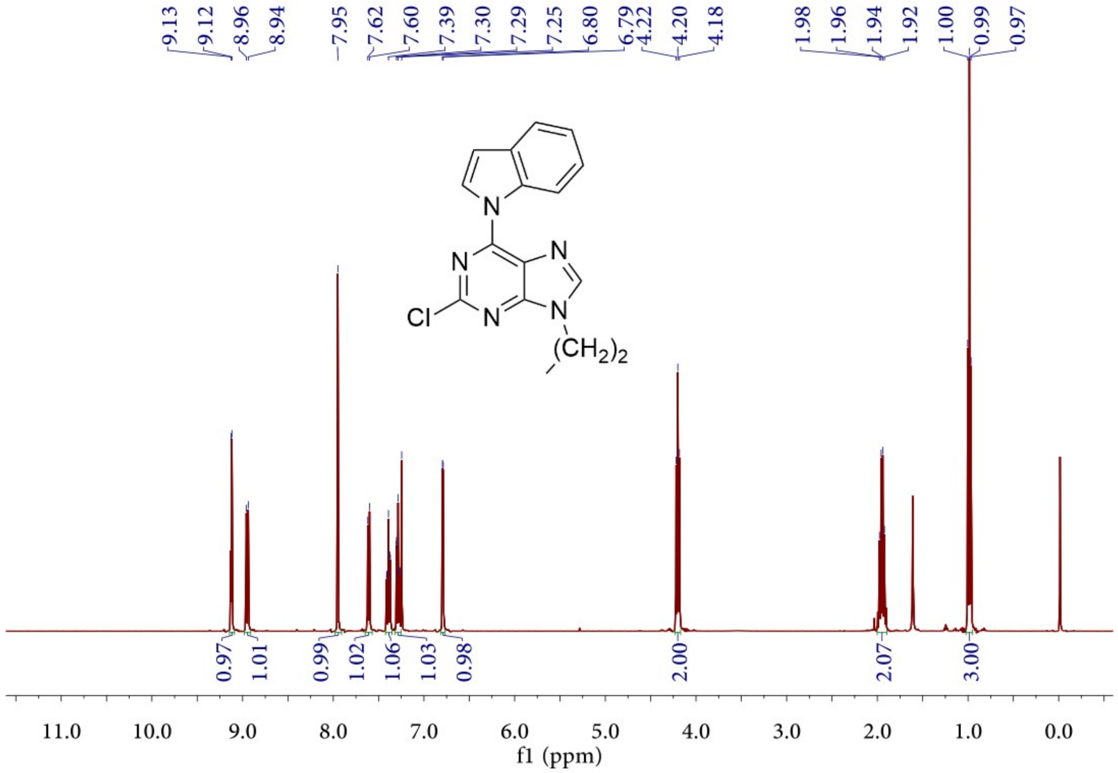

^{1}H NMR of Compound **3** in d^{6}-DMSO

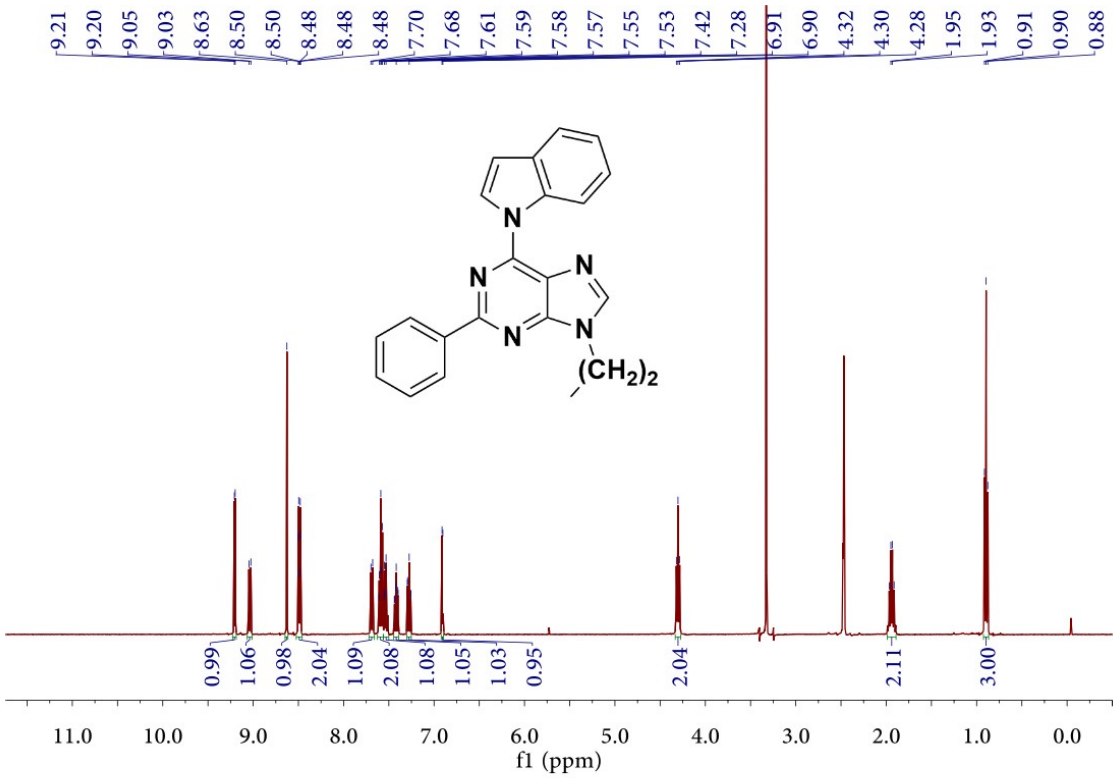

^{1}H NMR of **AIP** in d^{6}-DMSO

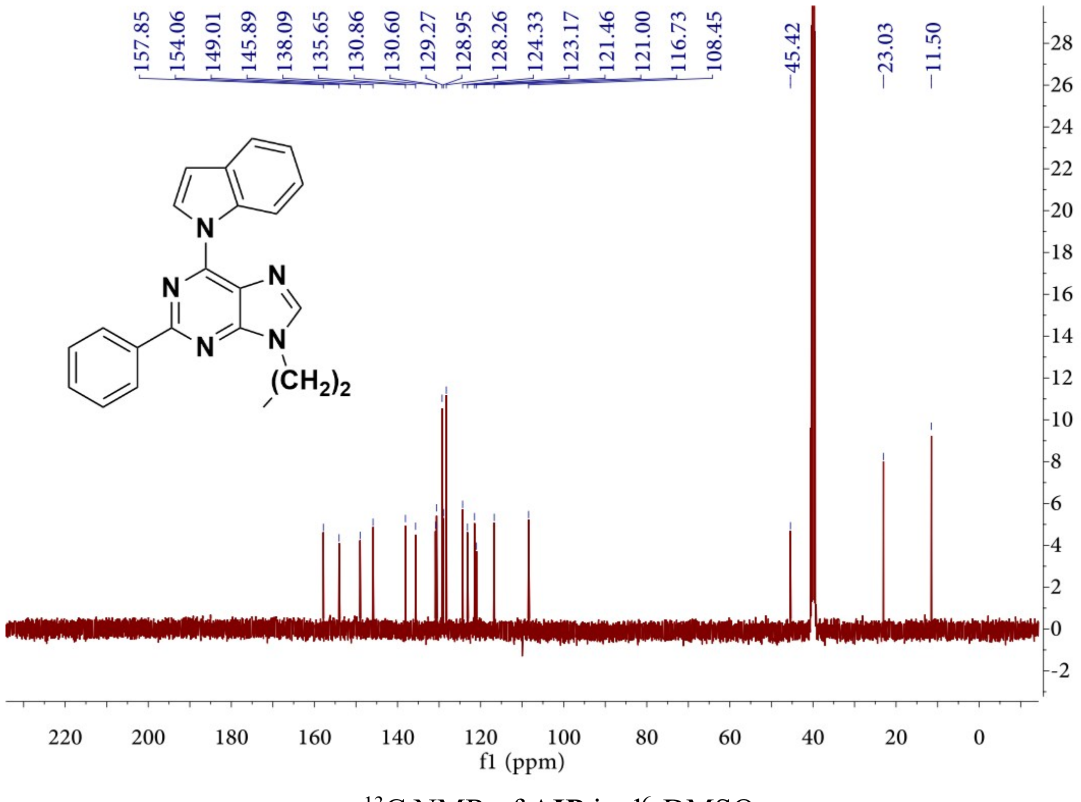

^{13}C NMR of **AIP** in d^{6}-DMSO

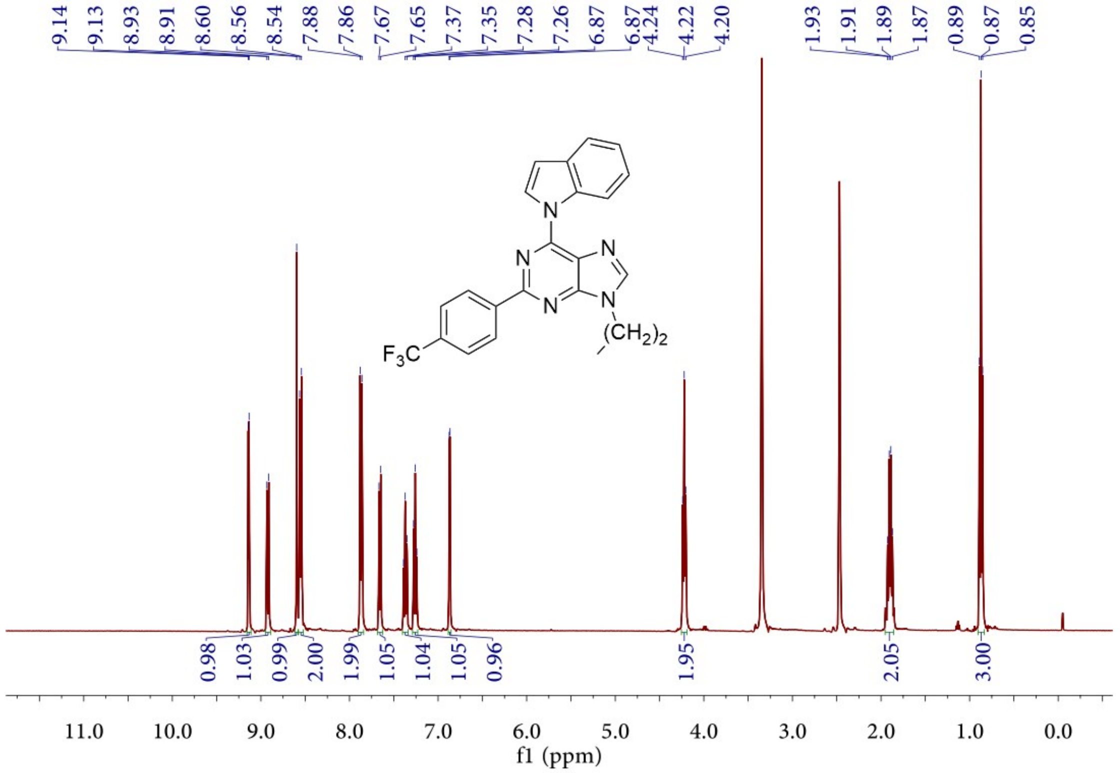

^{1}H NMR of **AIP-CF** in d^{6}-DMSO

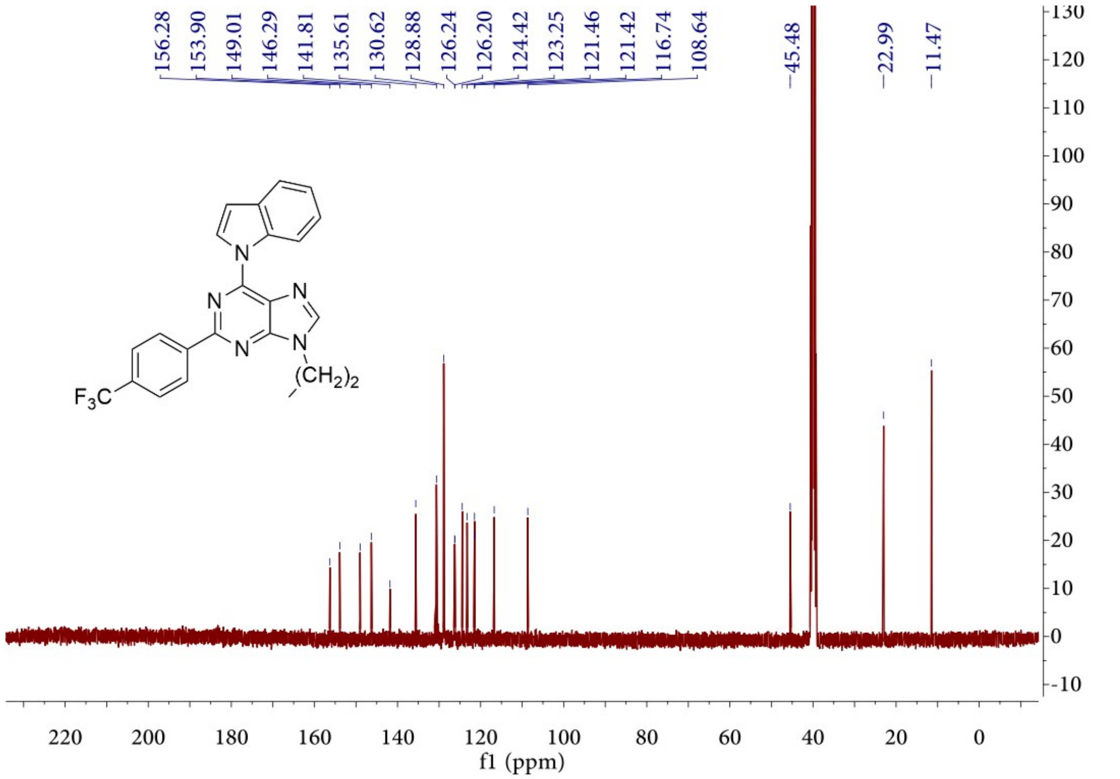

^{13}C NMR of **AIP-CF** in d^{6}-DMSO

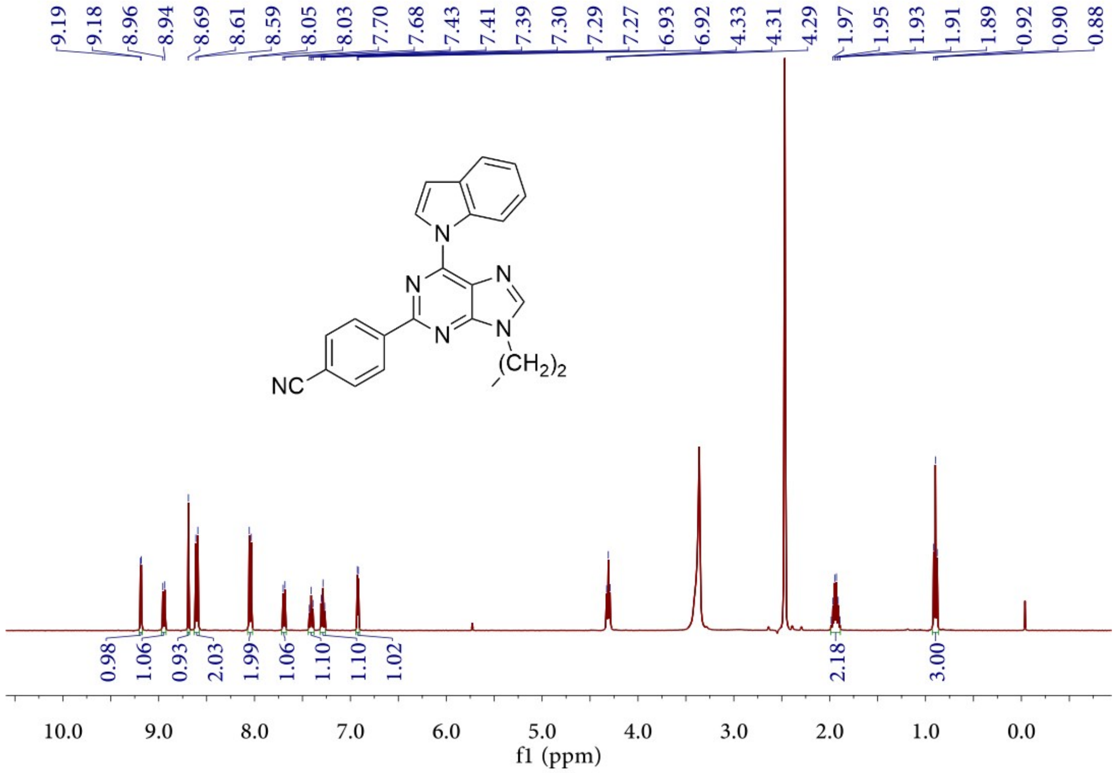

^{1}H NMR of **AIP-CN** in d^{6}-DMSO

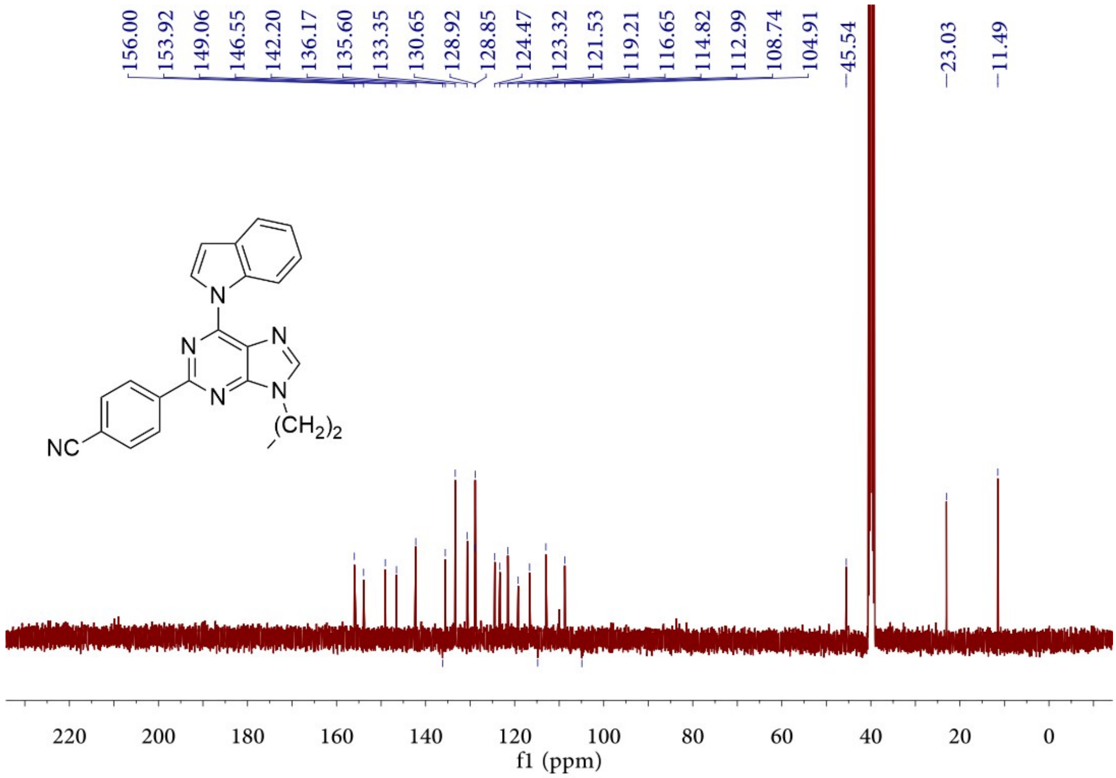

^{13}C NMR of **AIP-CN** in $CDCl_3$

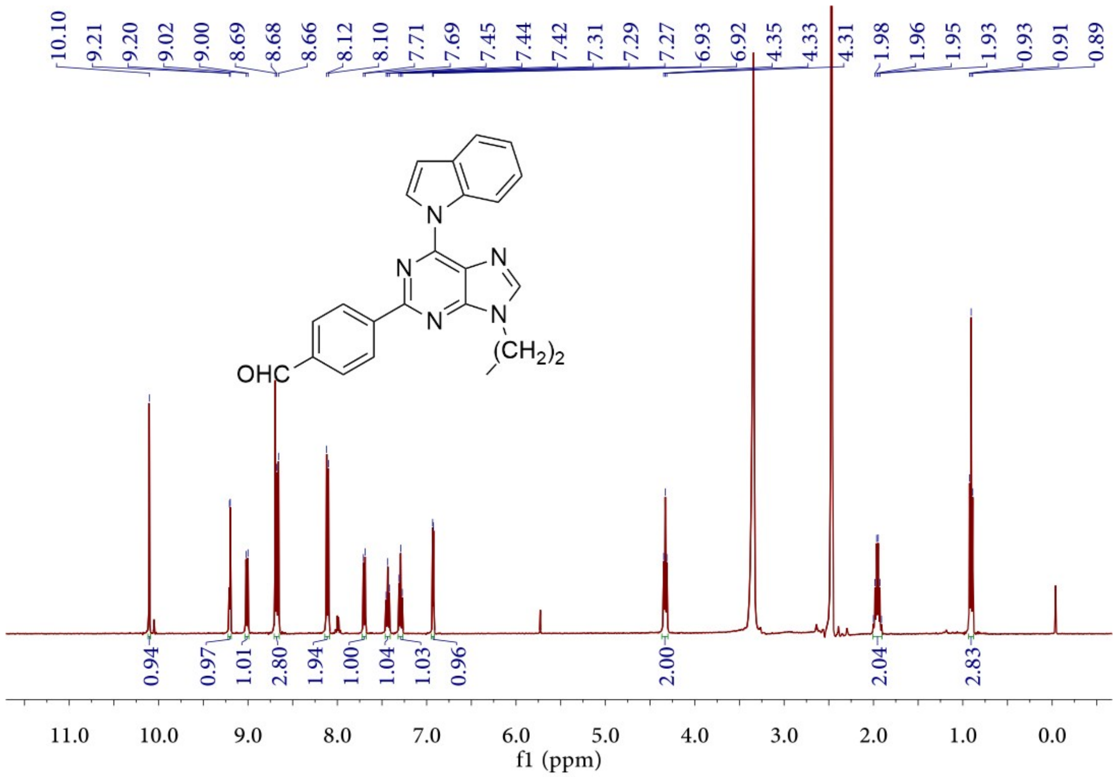

^{1}H NMR of **AIP-CHO** in d^{6}-DMSO

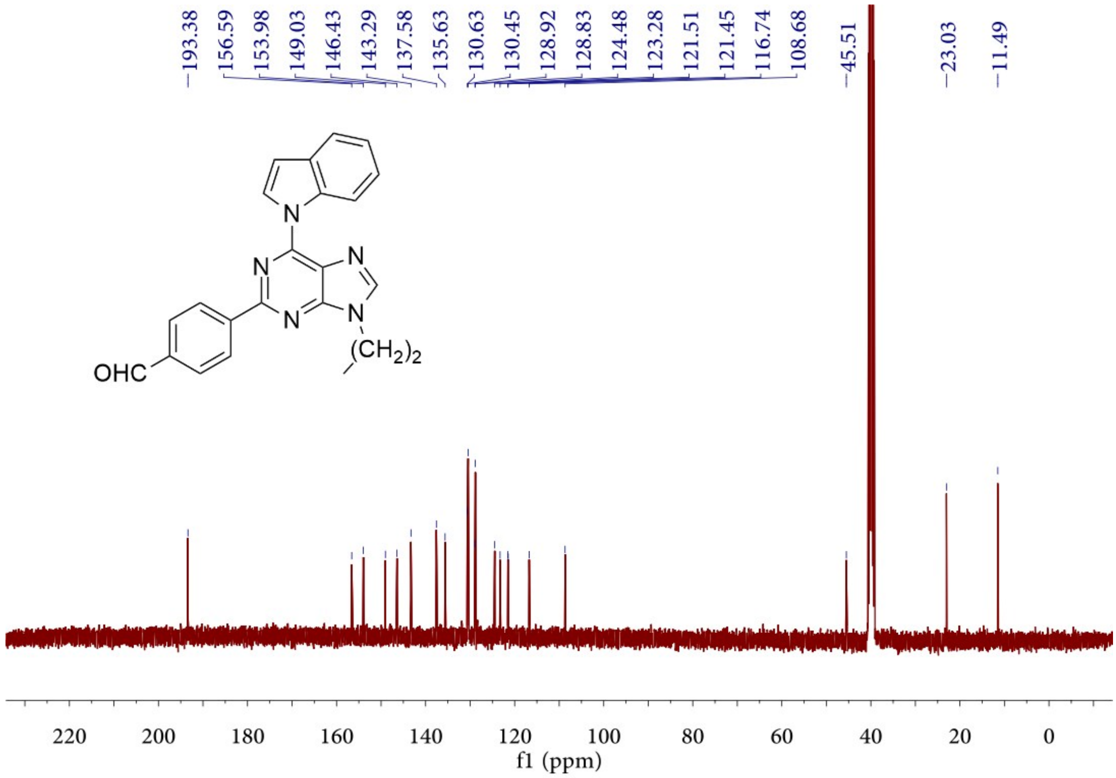

^{13}C NMR of **AIP-CHO** in d^{6}-DMSO

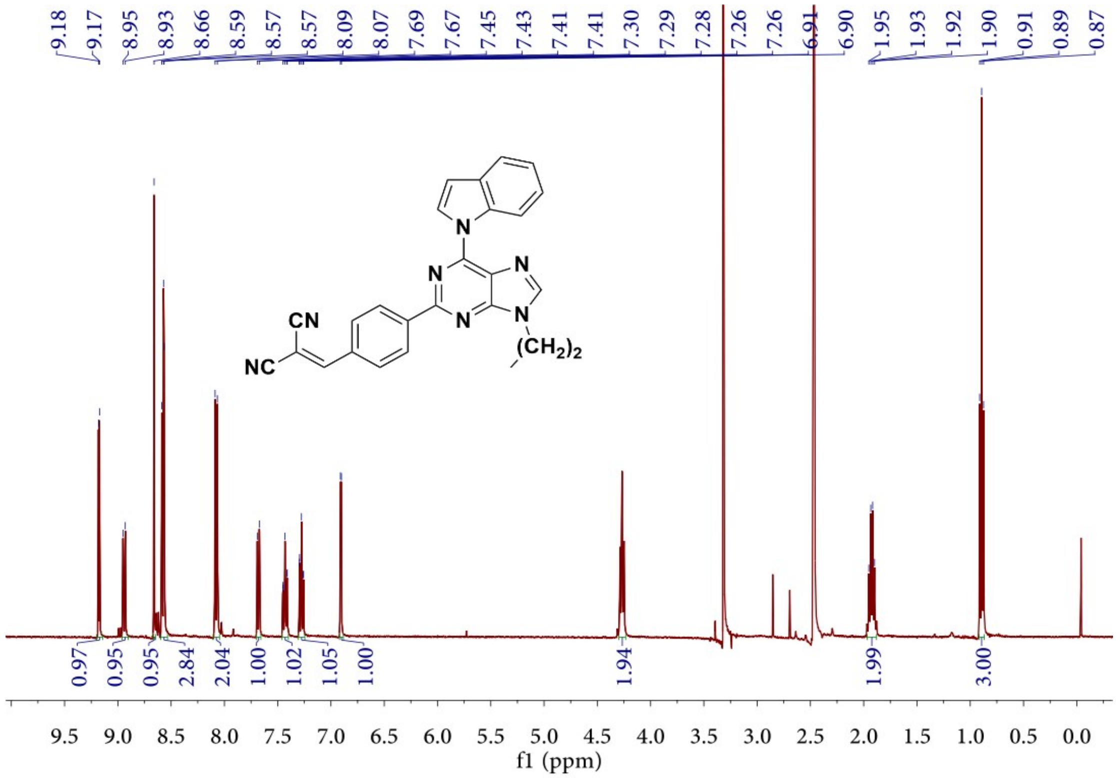

^{1}H NMR of **AIP-CN2** in d^{6}-DMSO

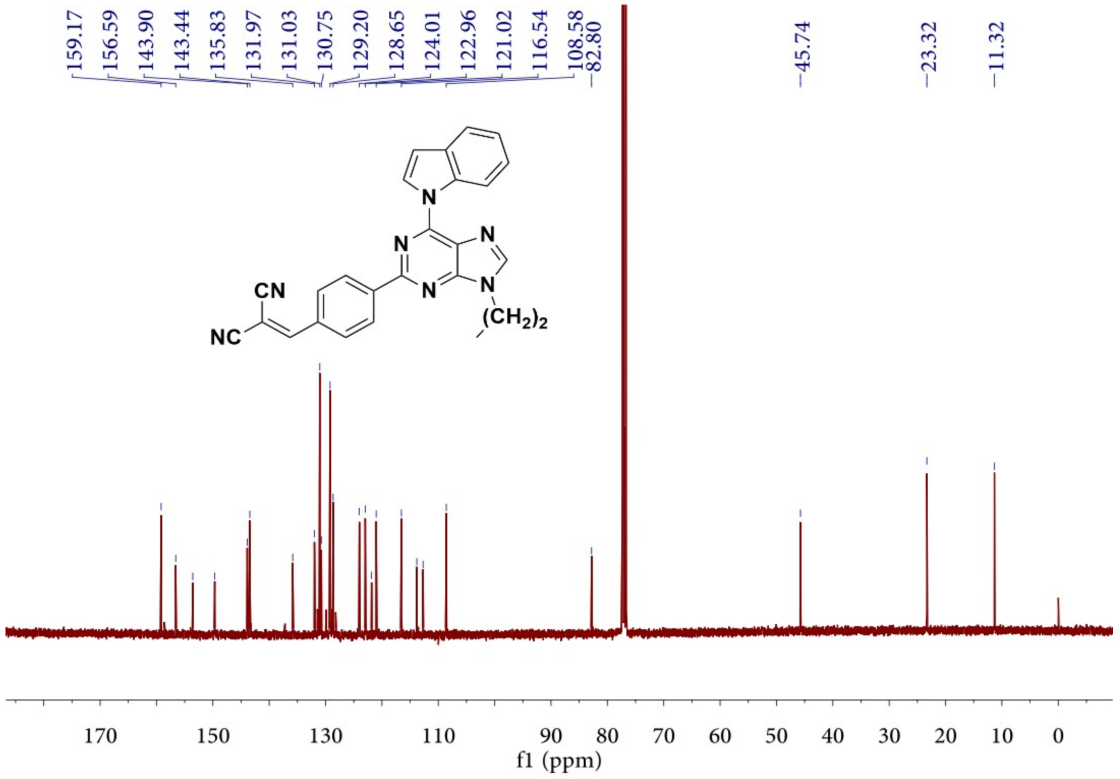

^{13}C NMR of **AIP-CN2** in $CDCl_3$

15. ESI-MS Data

AIP:

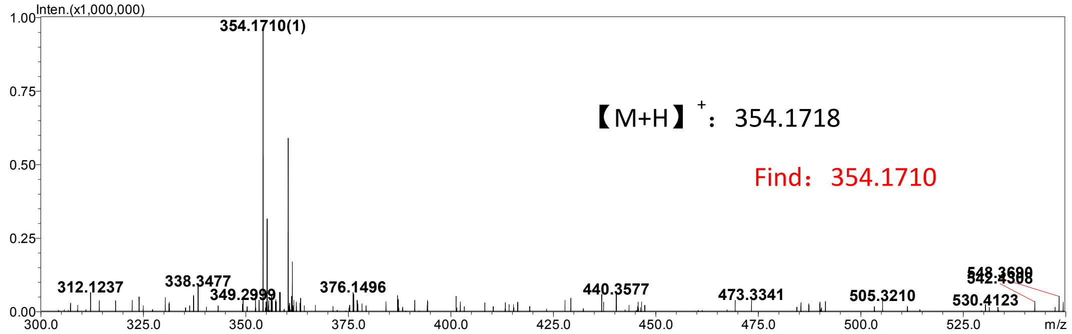

AIP-CF:

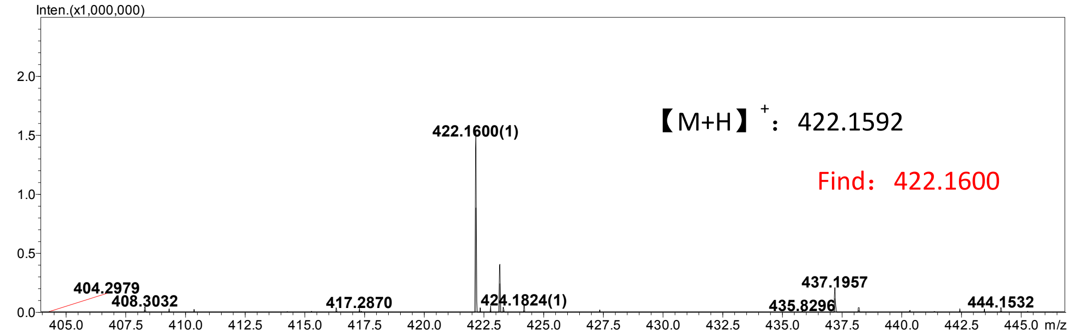

AIP-CN:

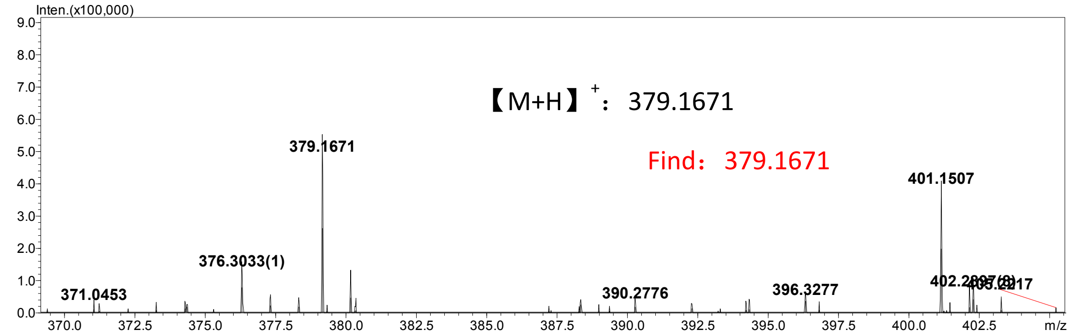

AIP-CHO:

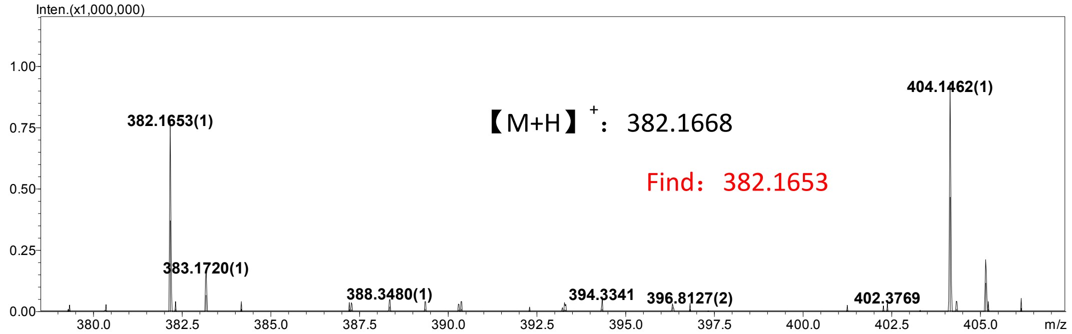

AIP-CN2:

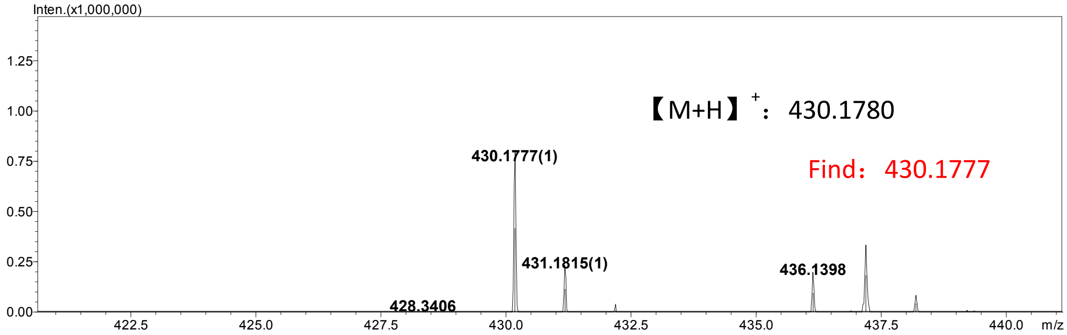
Inten.(x1,000,000)
1.25
1.00
0.75
0.50
0.25
0.00
【M+H】+：430.1780
Find：430.1777
430.1777(1)
431.1815(1)
436.1398
428.3406
422.5
425.0
427.5
430.0
432.5
435.0
437.5
440.0
m/z
